# We know very little about pollination in the *Platanthera* Rich (Orchidaceae: Orchidoideae)

**DOI:** 10.1002/ece3.11223

**Published:** 2024-04-10

**Authors:** Jasmine K. Janes, Genevieve E. van der Voort, Dezene P. W. Huber

**Affiliations:** ^1^ Biology Department Vancouver Island University Nanaimo British Columbia Canada; ^2^ Faculty of Environment University of Northern British Columbia Prince George British Columbia Canada; ^3^ IUCN, Species Survival Commission, Orchid Specialist Group

**Keywords:** bog orchid, hybridization, Insecta, pollination ecology, pollination networks, pollination syndrome, pollinator, reproduction

## Abstract

The *Platanthera* Rich. (Orchidoideae) comprise a speciose genus of orchids primarily in the northern hemisphere, with up to 200 known species worldwide. Individual species are known to self‐pollinate, but many rely on insect pollinators with characteristics such as floral color, timing of floral odor emissions, nectar rewards, and spur length associated with particular pollination syndromes. As with many orchids, some orchid–pollinator associations are likely highly co‐evolved, but we also know that some *Platanthera* spp. are the result of hybridization events, which implies a lack of pollinator fidelity in some cases. Some *Platanthera* spp. occur in large numbers which, coupled with the numerous *Platanthera*–pollinator systems, make them accessible as study species and useful for co‐evolutionary studies. Due to the likely effects of climate change and ongoing development on *Platanthera* spp. habitats, these orchids and their associated pollinators should be a focus of conservation attention and management. However, while there is a fairly substantial literature coverage of *Platanthera*–pollinator occurrence and interactions, there are still wide gaps in our understanding of the species involved in these systems. In this systematic review, we outline what is current knowledge and provide guidance on further research that will increase our understanding of orchid–insect co‐evolutionary relationships. Our review covers 157 orchid species and about 233 pollinator species interacting with 30 *Platanthera* spp. We provide analyses on aspects of these interactions such as flower morphology, known insect partners of *Platanthera* species, insect‐*Platanthera* specificity, pollination visitor timing (diurnal vs. nocturnal), floral rewards, and insect behavior affecting pollination outcomes (e.g., pollinia placement). A substantial number of *Platanthera* spp. and at least a few of their known pollinators are of official (IUCN) conservation concern – and many of their pollinators remain unassessed or even currently unknown – which adds to the urgency of further research on these co‐evolved relationships.

## Graphical abstract

This review of *Platanthera* pollination ecology shows that there are a number of orchid species in the group without pollinator information. Considerable knowledge gaps were identified even for those species where pollinator information has been reported, and these gaps impact our ability to appropriately manage orchid and pollinator populations, which may negatively effect conservation efforts. We provide a number of recommendations that can be applied broadly to a number of plant groups to further our understanding of pollination ecology.

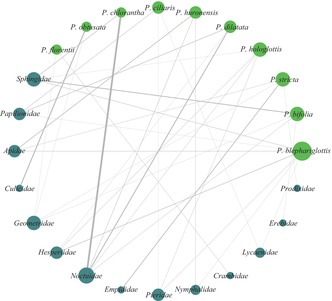

## 1 INTRODUCTION

Pollination services are crucial for the reproduction of most angiosperms. Over 87% of angiosperms are believed to be pollinated by animal vectors (Chupp et al., 2015), and it is estimated that we know of pairings between approximately 400,000 insect and 350,000 plant species (Schatz et al., 2020). Animal‐provisioned pollination can be more efficient and more specific than abiotic pollination (e.g., wind pollination), resulting in increased seed set, and contributes to floral diversification, variable gene flow, species isolation or co‐existence, and speciation. Thus, pollination ecology is a key area of study in plant–animal interaction research, with many models, hypotheses, and categorizations of interaction types.

A pollination syndrome is a particular suite of floral traits that adapts a flower for a specific group of pollinators or even a single, specialized pollinator. Thus, floral traits should adapt to the most efficient pollinator (Stebbins, 1970), driving convergent evolution of floral features in many plant species (Dellinger, 2020). Typically, syndromes will reflect floral trait evolution in response to a functional group of pollinators (e.g., moth, fly, and bird), and syndromes have often been used to predict pollinator groups (Ollerton et al., 2009). For example, white flowers with a nectar spur that produce scent at night might be expected to attract a nocturnal moth pollinator through a reward system. In fact, Charles Darwin made exactly this prediction when examining the long nectar spurs of the orchid (Orchidaceae) *Angraecum sesquipedale* (Arditti et al., 2012); the prediction was proven true with the later discovery of the sphinx moth, *Xanthopan morganii praedicta* (Arditti et al., 2012). In spite of widespread use as a research concept, pollination syndromes have been criticized for a lack of consistency in the floral traits used, issues with terminology, definitions of what constitutes a pollinator, and for oversimplification of complex interactions (Dellinger, 2020; Fenster et al., 2004; Ollerton et al., 2018).

Pollination syndromes provide a mechanistic link to help explain morphological variation arising from differences in male and female reproductive fitness and convergent evolution of floral traits when relying on similar pollinator groups. The Bateman principle (Arnold, 1994) suggests that female fitness is limited by ovule or seed production and that male fitness is limited by the ability to fertilize ovules (Catling & Catling, 1991; Chupp et al., 2015). Thus, floral traits that maximize pollen transfer should be under stronger selection pressure than traits that maximize pollen deposition and subsequent fertilization rates (Arnold, 1994). Applying the Bateman principle is challenging in plants because pollinators are often limited, observations of pollination and subsequent fertilization can be rare, and because of the sessile nature of plants, high rates of polyandry are assumed to be common (Tonnabel et al., 2019).

Orchids are renowned for their sheer diversity of floral forms, which can be directly related to the range of associated pollinators (e.g., birds, insects) (van der Cingel, 2001), different levels of pollinator specialization (e.g., generalist, specialist) (Argue, 2012), co‐evolutionary mosaics (Trunschke et al., 2019), and the diverse pollination systems that result (Boberg et al., 2014; Gaskett, 2011). Orchid pollination systems may use nectar rewards (e.g., *Spiranthes*) (Catling, 1983), provisioning of shelter (e.g., *Serapias*) (Vereecken et al., 2010), sexual deception (e.g., *Pterostylis*) (Phillips et al., 2014), food deception (e.g., *Orchis*) (Cozzolino et al., 2006), or be rewardless (e.g., Orchidinae members) (Vereecken et al., 2010).

In spite of high species richness, and some authors suggesting that orchids are over‐represented in the pollination literature compared to other plant groups (Peakall, 2007), there is still a great deal we do not know about orchid pollination (Schatz et al., 2020) and its role in the continued evolution of orchid–insect interactions. Given the diversity in orchid pollination, orchid–insect interactions represent useful models for understanding pollination ecology in other plant groups. In addition, high numbers of orchid species are at risk of extinction (Swarts & Dixon, 2009), there is widespread concern that pollinator populations are declining (Owens & Lewis, 2018; Young et al., 2017), and that climate change will lead to range shifts (Bedford et al., 2012; Gómez‐Ruiz & Lacher, 2019) and phenological asynchronies (Adedoja et al., 2020; Slominski & Burkle, 2021) that will further impact plant–insect interactions among the Orchidaceae and beyond. Thus, understanding orchid–insect relationships is increasingly important not only for orchid conservation but also for developing and applying an understanding of pollination networks and biodiversity conservation in a variety of plant communities.

*Platanthera* Rich. (Orchidoideae) is the largest genus of terrestrial orchids in the Northern Hemisphere (Hapeman & Inoue, 1997; Sears, 2008; Wettewa & Wallace, 2021). Estimates range from 85 (Hapeman & Inoue, 1997) to 200 species (Eum & Lee, 2012; Sears, 2008), and the genus is considered particularly diverse in North America (Wettewa & Wallace, 2021). *Platanthera* spp. are recognized by their elongate tubers, nectar‐containing floral spurs, and lack of a prominent stigmatic process (Sears, 2008). *Platanthera* spp. exhibit diverse floral morphology and several pollination syndromes have been characterized for the genus (Argue, 2012; Hapeman & Inoue, 1997). The diversity of floral features, coupled with the frequent reports of hybridization among certain species (e.g., *P. bifolia* × *P. chlorantha* (Esposito et al., 2017), and *P. dilatata* × *P. aquilonis* (Wallace, 2004)), make *Platanthera* spp. an excellent study system for advancing our understanding of pollination ecology, the application of pollination syndromes, plant‐insect interactions, reproductive barriers, floral evolution, and general patterns of orchid and pollinator diversity. However, our advances in these areas are often hampered by orchid species delineations, which rely on some of the same pollination syndrome characters that are subject to pollinator‐driven selection pressures (Hapeman & Inoue, 1997), a lack of natural historical observations, and the need for targeted research to distinguish non‐pollinating floral visitors from pollinators.

Because it is a widespread, species‐rich group that seems particularly amenable to ecological and evolutionary research questions, we were interested in understanding the current state of the *Platanthera* pollinator literature, with a particular focus on the accuracy of currently applied pollination syndromes, and what knowledge gaps remain. Reviews by van der Cingel (2001), Argue (2012), and Pace (2020) each covered a subset of the species present within the genus because they focused on different geographic regions and highlighted different aspects of pollination. The review by Pace (2020) focused on systematics of orchids in northeastern North America and only superficially mentioned pollination ecology. Whereas the reviews by van der Cingel (2001) and Argue (2012) presented broad overviews of pollination systems (e.g., rewarding or not; pollination syndromes) but lacked specific detail regarding pollinator species, behavior, floral trait variation, and reproductive success. Thus, a comprehensive review specifically of the *Platanthera*, and their pollinators, across their geographic range has not been conducted. This review spans all of the current, accepted, reported species from across the entire geographic range, which includes every continent except Antarctica and South America.

## 2 METHODS AND MATERIALS

### 2.1 Selection of species and sources of information

**FIGURE 1:**
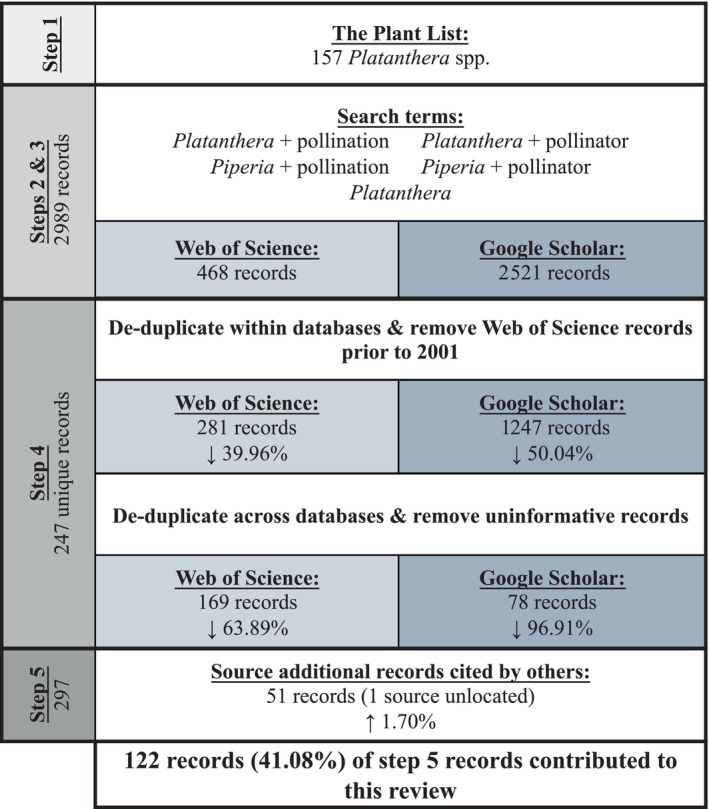
Workflow of search and filter steps to obtain literature records used in this review.

Our approach consisted of five steps (see Figure 1). First, we obtained a complete list of commonly accepted species within the *Platanthera* from The Plant List on 10th May 2022 (http://www.theplantlist.org/tpl1.1/record/kew‐157023). The accepted names were used to focus our readings and data compilation as orchid taxonomy is quite dynamic (Chase et al., 2015; Dressler, 1993; Janes & Duretto, 2010). Second, Web of Science was used to perform a primary literature search using the following combinations of search terms across all fields: (1) *Platanthera* + pollination; (2) *Platanthera* + pollinator; (3) *Piperia* + pollination; (4) *Piperia* + pollinator; and (5) *Platanthera*. We included *Piperia* spp. (the rein orchids) in our search terms as the genus was not officially amalgamated with the *Platanthera* until 2009 (Bateman et al., 2009). Third, we performed a complimentary literature search, using Publish or Perish v7.33.3388 (Harzing, 2007), that cross‐referenced Google Scholar. Given the number of records that Google Scholar searches can generate and the dynamic nature of its search algorithms (Giustini & Boulos, 2013), we restricted our search dates to 2001–2022 across titles and abstracts, to account for the fact that one of the last reviews of *Platanthera* spp. pollination ecology was performed in 2001 (van der Cingel, 2001). While Argue (2012) did not appear to add substantial new information for the *Platanthera*, and often cited van der Cingel (2001). Fourth, we manually filtered the combined results from Web of Science and Google Scholar. These filters included: de‐duplicated records within database searches, removing Web of Science records prior to 2001, de‐duplicating records across database searches, removing non‐English articles for which a translation could not be found, and removing uninformative records based on title and abstract. For example, several papers describing complete chloroplast genomes for *Platanthera* spp. did not contain useful pollinator or floral visitor information. Fifth, because many sources cited others for their information, we obtained as many of the original articles as possible using web sources or institutional interlibrary loans where necessary to verify the information.

### 2.2 Data collection and categories

While collecting information on *Platanthera*–insect associations, we collected data on *Platanthera* morphology, distribution, scent, and reproductive success when available in the literature. Full details of data collected can be found in Appendix S1. Given the inconsistency of information provided across sources, we applied broad categories to some elements of the data. For example, orchid species' distributions were determined at a continental scale. *Platanthera* spp. were grouped according to descriptions of several floral traits believed to be useful in characterizing pollinator syndromes and explaining reproductive isolation among sympatric and/or sister taxa (Boberg et al., 2014; Brand et al., 2020; Schiestl & Schlüter, 2009) – flower color, spur length, labellum type, and scent. Morphological categories were determined based on common language or characters provided by authors. For example, many authors applied the term ‘short’ to spurs measuring 10 mm or less. While such categories are broad, and somewhat subjective, similar approaches have been used by others (Hapeman & Inoue, 1997; Schiestl & Schlüter, 2009), and provided insight for our categorization.

### 2.3 Statistical analysis and data visualization

Means were calculated for a number of categories including insect taxa per orchid species, and orchid species. A pollinator sharing index (Schiestl & Schlüter, 2009) was calculated for each pollinator species (index = (orchid spp. sharing the pollinator – 1)/(total number orchid spp. – 1)). Index values range between 0 and 1, with 0 indicating no pollinator sharing and 1 indicating complete overlap in pollinators. A two‐tailed Fisher Exact test was performed in Microsoft Excel to assess independence among flower color and insect activity time. Venn diagrams were made in R 4.2.2 (R Core Team, 2021) using packages ggvenn (Yan, 2021) and ggVennDiagram (Gao et al., 2021). Network analysis figures were produced with igraph (Csárdi & Nepusz, 2006).

## 3 RESULTS

### 3.1 Literature search results

Web of Science searches yielded the following numbers of records: (1) *Platanthera* + pollination = 123; (2) *Platanthera* + pollinator = 92; (3) *Piperia* + pollination = 1; (4) *Piperia* + pollinator = 2; and (5) *Platanthera* = 338. Google Scholar yielded: (1) *Platanthera* + pollination = 997; (2) *Platanthera* + pollinator = 803; (3) *Piperia* + pollination = 285; (4) *Piperia* + pollinator = 282; and (5) *Platanthera* = 154. Thus, Web of Science generated 468 records, and Google Scholar returned 2521 records for a combined total of 2989 non‐unique records (Figure 1). After de‐duplicating records and removing Web of Science records prior to 2001, 60.04% (281) and 49.46% (1247) records were retained from Web of Science and Google Scholar, respectively. De‐duplicating records across databases and removing uninformative records yielded 36.11% (169) and 3.09% (78) records for Web of Science and Google Scholar, respectively. In total, 247 unique records, representing 8.26% of the total records retrieved, were considered suitable for further investigation.

Of the 247 unique records, we were unable to locate one full‐text from the Google Scholar search (i.e., Shadge et al., 2019 – see Appendix S1). We sourced and read the remaining 246 records; 34.62% (71) literature sources were useful in contributing pollinator or floral visitor information. An additional 51 papers were sourced and read because they were cited by others, bringing the total number of unique literature sources to 297. Thus, 122 (41.08%) of these 297 literature sources contributed to this review. Refer to Appendix S1 for full details of literature sources that contributed to this review. We were unable to acquire full‐text copies of some literature sources cited by others. Similarly, we were unable to verify some information sourced from personal communications. These instances are highlighted in Appendix S1.

### 3.2 General *Platanthera* information and trends

**FIGURE 2:**
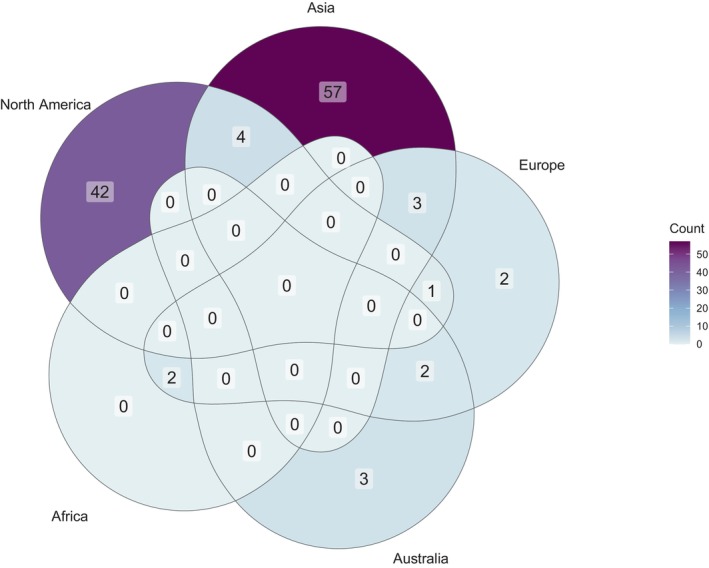
Venn diagram illustrating the continental‐level distribution of *Platanthera* species.

The Plant List returned 157 accepted *Platanthera* spp., of which 18 (11.46%) are recognized hybrids, and 20 (12.74%) have been assessed for an IUCN status (Table 1). One species, *P. clavellata*, was cited as being definitively autogamous or lacking any pollinator (Catling, 1983). In terms of continental distribution, 64 (40.76%) species were reported for Asia, 47 (29.94%) for North America, 10 (6.37%) for Europe, five (3.18%) for Australia, and two (1.27%) for Africa (Figure 2). No species were reported in the literature from South America. While there are records of *Platanthera* spp. from South America on iNaturalist, it is possible that these are misidentifications. A striking 41 species (26.11%) are missing distribution information based on the sources of information we read.

**TABLE 1:** Accepted *Platanthera* species with their respective distributions, morphologies, and ecological information.

Orchid species	Literature source	IUCN status	Continent	Flowering period	Labellum type	Dominant flower color	Nectar	Spur category	Scent
*P. × andrewsii* (M.White) Luer	Correll (1939)		North America	Jun–Sep	Fringed	White‐pink		M/L	
*P. × apalachicola* P.M.Br. & S.L.Stewart	Brown and Stewart (2003), Brown (2004)		North America	Jun–Sep	Fringed	Orange‐yellow			
*P. × beckneri* P.M.Br.	van der Cingel (2001)		North America		Fringed	White‐orange			
*P. × bicolor* (Raf.) Luer	Pace (2020)		North America		Fringed	Yellow‐cream			
*P. × canbyi* (Ames) Luer	Kiefer (2009)		North America		Fringed	Orange‐yellow			
*P. × channellii* Folsom	Brown (2004)		North America		Fringed	Orange			
*P. × correllii* W.J.Schrenk	Brown (2010)		North America		Entire	Green‐yellow			
*P. × enigma* P.M.Br.	Brown (2008)		North America		Fringed	Pink‐purple		L	
*P. × hollandiae* Catling & Brownell	Catling and Brownell (1999)		North America		Fringed	White‐green		L	
*P. × hybrida* Brügger	Efimov (2016)		Europe	May	Entire	White‐green			
*P. × inouei* Efimov	Efimov (2016)		Asia						
*P. × lueri* P.M.Br.	Brown (2006)		North America		Fringed	Orange‐yellow			
*P. × mixta* Efimov	Efimov (2016)		Asia		Entire	White‐green			
*P. × okubo‐hachijoensis* K.Inoue	Efimov (2016)		Asia		Entire	Green			
*P. × ophryotipuloides* K.Inoue	Efimov (2016)		Asia						
*P. × osceola* P.M.Br. & S.L.Stewart	Brown and Stewart (2003), Brown (2004)		North America						
*P. × reznicekii* Catling, Brownell & G.Allen	Catling et al. (1999)		North America	July	Fringed	White‐pink		M/L	
*P. × vossii* F.W.Case			North America	Jun–Aug		White‐green			
*P. albomarginata* Kraenzl.									
*P. algeriensis* Batt. & Trab.	Bateman et al. (2012), Efimov (2016)		Africa, Europe	Apr–Jul	Entire	Green			
*P. alpinipaludosa* P.Royen	Efimov (2016)		Australia (New Guinea)						
*P. amabilis* Koidz.	Efimov (2016)		Asia	Jun–Aug	Entire	Green		S/M	
*P. angustata* (Blume) Lindl.	Efimov (2016)		Asia		Entire	Green‐white		S/M	No
*P. angustilabris* Seidenf.	Efimov (2016), MingXu et al. (2018)		Asia	Sep–Oct	Entire	Yellow‐green		L	
*P. aquilonis* Sheviak	Hapeman and Inoue (1997), Sears (2008)		North America		Entire	Green			No
*P. arcuata* Lindl.									
*P. arfakensis* Renz									
*P. azorica* (Schltr.)	Bateman et al. (2012), Efimov (2016)		Europe	Jun–Jul		Green	Y		Night
*P. bakeriana* (King & Pantl.) Kraenzl.	Efimov (2016)		Asia	Jun–Sep	Entire	Green‐yellow		S/M	Yes
*P. bhutanica* K.Inoue									
*P. biermanniana* (King & Pantl.) Kraenzl.									
*P. bifolia* (L.) Rich.	Bateman et al. (2012), Efimov (2016), Trunschke et al. (2020)		Europe, Asia	May–Aug	Entire	White‐green	Y	M/L/VL	Night
*P. blephariglottis* (Willd.) Lindl.	Smith and Snow (1976), Catling and Catling (1991), van der Cingel (2001)		North America	Jul–Aug	Fringed	White	Y	L	Day/night
*P. blumei* Lindl.	Efimov (2016)		Asia		Entire	Green		S/M	
*P. boninensis* Koidz.	Inoue (1983), Efimov (2016)		Asia	Mar–Jun	Entire	White		M/L	
*P. borneensis* (Ridl.) J.J.Wood									
*P. brevicalcarata* Hayata	Eum & Lee, 2012, Efimov (2016)		Asia	May–Jul	Entire	White		S	
*P. brevifolia* (Greene) Senghas	Efimov (2016)				Entire	White‐green – achlorophyllous		S/M	
*P. calderoniae* López‐Ferr. & Espejo									
*P. chapmanii* (Small) Luer	Poff et al. (2016)		North America	Jul–Aug	Fringed	Orange		S/M	
*P. chiloglossa* (Tang & F.T.Wang) K.Y.Lang									
*P. chlorantha* (Custer) Rchb.	Bateman et al. (2012), Efimov (2016), Steen et al. (2019)		Europe, Asia	Apr–Aug	Entire	White‐green	Y	M/L/VL	Day/night
*P. chorisiana* (Cham.) Rchb.f.	Efimov (2016)		Asia, North America	Jun–Aug	Entire	Green‐white		S	
*P. ciliaris* (L.) Lindl.	Smith and Snow (1976), Chupp et al. (2015)		North America	August	Fringed	Orange	Y	L	
*P. fornicate* (Michx.) Luer	Hapeman and Inoue (1997)		North America		Entire	Green		S/M	
*P. clavigera* Lindl.									
*P. colemanii* (Rand.Morgan & Glic.) R.M.Bateman									
*P. contigua* Tang & F.T.Wang	Efimov (2016), Liden and Bharali (2019)		Asia		Entire	Green‐white		S	
*P. convallariifolia* (Fisch. Ex Lindl.) Lindl.	Efimov (2016)		North America, Asia	Jun–Aug	Entire	Green‐yellow		S	
*P. cooperi* (S.Watson) R.M.Bateman									
*P. crassinervia* (Ames & C.Schweinf.) J.J.Sm.									
*P. cristata* (Michx.) Lindl.	Hapeman and Inoue (1997)		North America		Fringed	Orange		S	
*P. cumminsiana* (King & Pantl.) Renz	Rai et al. (2015)		Asia	Jul–Sep	Entire	Green‐yellow		S	
*P. curvata* K.Y.Lang									
*P. damingshanica* K.Y.Lang & H.S.Guo									
*P. deflexilabella* K.Y.Lang	Efimov (2016)		Asia	Jun–Aug	Entire	Yellow‐green		S	
*P. densa* Freyn	Efimov (2016)		Asia	May–Jul	Entire	White		M/L	
*P. devolii* (T.P.Lin & T.W.Hu) T.P.Lin & K.Inoue	Efimov (2016)		Asia	Jun–Sep	Entire	Yellow‐green		S/M	
*P. dilatata* (Pursh) Lindl. Ex L.C.Beck	Boland (1993), Efimov (2016)		North America, Asia	May–Aug	Entire	White	Y	S	Day/night
*P. dyeriana* (King & Pantl.) Kraenzl.	Singh et al. (2014), Efimov (2016)		Asia	Jul–Aug	Entire	Green‐yellow		S/M	
*P. edgeworthii* (Hook.f. ex Collett) R.K.Gupta									
*P. elegans* Lindl.	Ackerman (1977)				Entire	White‐green		S/M	
*P. elliptica* J.J.Sm.	Efimov (2016)		Asia		Entire	Green		S/M	
*P. fornicat* (Rydb.) R.M.Bateman	J. K. Janes (2021) (personal observation)		North America	Jun–Jul	Entire	Green		S/M	
*P. ephemerantha* R.M.Bateman	J. K. Janes (2021) (personal observation)		North America	Jun–Jul	Entire	White‐green			
*P. epiphytica* Aver. & Efimov	Efimov (2016)		Asia		Entire	Green‐yellow		M	
*P. exelliana* Soó	Efimov (2016)		Asia	Jul–Sep	Entire	Green		S	Yes
*P. finetiana* Schltr.	Efimov (2016)		Asia	Jun–Aug	Entire	White‐green		M/L	
*P. flava* (L.) Lindl.	van der Cingel (2001), Efimov (2016)		North America	Mar–Oct	Entire	Green	Y	S/M	
*P. florentia* Franch. & Sav.									
*P. florentii* Franch. & Sav.	Suetsugu and Hayamizu (2014), Efimov (2016)		Asia	Aug–Sep	Entire	Green		S/M	
*P. fornicata* (Bab.) Buttler	Bateman et al. (2012)		Europe			Green			
*P. fuscescens* (L.) Kraenzl.	Hapeman and Inoue (1997), Efimov (2016)		Asia	May–Jul	Entire	Green‐yellow		S/M	
*P. grandiflora* (Bigelow) Lindl.	Stoutamire (1974), Hapeman and Inoue (1997), van der Cingel (2001)		North America	June	Fringed	Purple‐pink	Y	M/L/VL	Day
*P. handel‐mazzettii* K.Inoue	Efimov (2016)		Asia	Jul–Sep	Entire	Green		S	
*P. herminioides* Tang & F.T.Wang									
*P. heyneana* Lindl.									
*P. holmboei* H.Lindb.	Bateman et al. (2012)		Europe			Green			
*P. hologlottis* Maxim.	Efimov (2016), Hattori et al. (2020)		Asia	Jun–Aug	Entire	White	Y	S/M/L	Yes
*P. hookeri* (Torr.) Lindl.	Boland (1993), Reddoch and Reddoch (2007)		North America	May–Jul	Entire	Green‐yellow		M	
*P. huronensis* Lindl.	Boland (1993), Sears (2008), Kropf (2015)		North America		Entire	Green‐white		S/M	Yes
*P. hyperborea* (L.) Lindl.	Lojtnant and Jacobsen (1976), Hapeman and Inoue (1997), Kropf (2015)		Europe, North America, Asia		Entire	Green	Y/N	S	Night/no
*P. iinumae* Makino	Efimov (2016)		Asia	Jul–Aug	Entire	Green‐white		S	
*P. integra* (Nutt.) A.Gray ex L.C.Beck	Hapeman and Inoue (1997)		North America		Entire	Yellow		S	
*P. integrilabia* (Correll) Luer	Zettler et al. (1996)				Entire	White	Y	L/VL	Yes
*P. japonica* (Thunb.) Lindl.	Efimov (2016)		Asia	Jun–Aug	Entire	White	Y	L/VL	
*P. kinabaluensis* Kraenzl. ex Rolfe	Efimov (2016)		Asia		Entire	Green‐yellow		S/M	Yes
*P. komarovii* Schltr.	Efimov (2016)		Asia	Jun–Aug	Entire	Green‐yellow		M	
*P. kuenkelei* H.Baumann	Bateman et al. (2012)		Africa						
*P. kwangsiensis* K.Y.Lang	Efimov (2016)		Asia	May–Aug	Entire	White		S	
*P. lacera* (Michx.) G.Don	Hapeman and Inoue (1997), Little et al. (2005)		North America	June	Fringed	White‐green		M	
*P. lancilabris* Schltr.									
*P. latilabris* Lindl.									
*P. leptocaulon* (Hook.f.) Soó	Efimov (2016)		Asia	Jun–Sep	Entire	Green‐yellow		M	
*P. leptopetala* (Rydb.) R.M.Bateman									
*P. leucophaea* (Nutt.) Lindl.	Hapeman and Inoue (1997), Thixton (2017)		North America		Fringed	White	Y	L/VL	Night
*P. likiangensis* Tang & F.T.Wang									
*P. limosa* Lindl.									
*P. longibracteata* Lindl.	Efimov (2016)		Asia	Jul–Sep	Entire	White‐green		S	
*P. longicalcarata* Hayata	Efimov (2016)		Asia		Entire	Green			
*P. longifolia* A.Rich. & Galeotti									
*P. longiglandula* K.Y.Lang	Efimov (2016)		Asia	Jun–Sep	Entire	Green‐yellow		S/M	
*P. mandarinorum* Rchb.f.	Efimov (2016)		Asia	Apr–Jul	Entire	Green‐yellow		M/L/VL	
*P. maximowicziana* Schltr.	Efimov (2016)		Asia	Jun–Sep	Entire	Green		S/M	
*P. michaelii* (Greene) R.M.Bateman									
*P. micrantha* (Hochst.) Schltr.	Bateman et al. (2012)		Europe	May–Jul		Green	Y		Night
*P. minor* (Miq.) Rchb.f.	Efimov (2016)		Asia	May–Aug	Entire	Yellow‐green		S/M	
*P. minutiflora* Schltr.	Efimov (2016)		Asia	Jun–Aug	Entire	White		S	
*P. neottianthoides* (Tang & F.T.Wang) R.M.Bateman & P.J.Cribb									
*P. nivea* (Nutt.) Luer	Hapeman and Inoue (1997)		North America			White			
*P. nubigena* A.Rich. & Galeotti									
*P. obtusata* (Banks ex Pursh) Lindl.	Efimov (2016), Lahondère et al. (2020)		North America	Jun–Aug	Entire	Green	Y	S	Yes
*P. okuboi* Makino	Efimov (2016)		Asia	May–Jun	Entire	White		L/VL	
*P. ophiocephala* (W.W.Sm.) Tang & F.T.Wang									
*P. ophrydioides* F.Schmidt	Efimov (2016)		Asia	Jun–Aug	Entire	Green		S/M	
*P. orbiculata* (Pursh) Lindl.	Hapeman and Inoue (1997)		North America		Entire	Green‐white		M/L/VL	
*P. orbiculata* var. *macrophylla* (Goldie) Luer	Hapeman and Inoue (1997)		North America			Green‐white			
*P. oreophila* Schltr.	Efimov (2016)		Asia	Jul–Sep	Entire	Green		M	
*P. pachycaulon* (Hook.f.) Soó	Rai et al. (2014)		Asia	Jun–Jul	Entire	Green		S	
*P. pallida* P.M.Br.	Brown (2008)		North America		Fringed	White‐cream			
*P. papuana* Schltr.	Efimov (2016)		Australia (New Guinea)		Entire	Green		M	
*P. peichatieniana* S.S.Ying	Efimov (2016)		Asia	October		Green‐white			
*P. peramoena* A.Gray	Hapeman and Inoue (1997)		North America		Entire	Purple		L	Yes
*P. praeclara* Sheviak & M.L.Bowles	Hapeman and Inoue (1997), van der Cingel (2001), Biederman et al. (2018), Travers et al. (2018)		North America	Jun–Jul	Fringed	White	Y	VL	Night
*P. psycodes* (L.) Lindl.	Stoutamire (1974), Hapeman and Inoue (1997), van der Cingel (2001)		North America	June	Fringed	Purple	Y	M	Day/night
*P. purpurascens* (Rydb.) Sheviak & W.F.Jenn.									
*P. replicata* (A.Rich.) Ackerman									
*P. robinsonii* J.J.Sm.	Efimov (2016)		Asia		Entire	Green		S	
*P. roseotincta* (W.W.Sm.) Tang & F.T.Wang	Efimov (2016)		Asia	JunvSep	Entire	White‐yellow		S	
*P. sachalinensis* F.Schmidt	Suetsugu and Hayamizu (2014), Efimov (2016)		Asia	Jul–Aug	Entire	White‐green		S/M	
*P. saprophytica* J.J.Sm.	Efimov (2016)		Asia		Entire	White – achlorophyllous		S	
*P. shriveri* P.M.Br.	Brown et al. (2008)		North America	Jul–Aug	Fringed	Purple		M/L/VL	
*P. sikkimensis* (Hook.f.) Kraenzl.	Efimov (2016)		Asia	Jun–Sep	Entire	Green		S/M	
*P. singgalangensis* (J.J.Sm.) Efimov									
*P. sinica* Tang & F.T.Wang									
*P. sonoharae* Masam.	Efimov (2016)		Asia	Sep–Dec	Entire	Yellow‐green		S	
*P. sparsiflora* (S.Watson) Schltr.	Kipping (1971)		North America	Jun–Jul	Entire	Green	Y	S	Night
*P. stapfii* Kraenzl. ex Rolfe	Efimov (2016)		Asia		Entire	Green		S/M	
*P. stenantha* (Hook.f.) Soó	Efimov (2016)		Asia	Jul–Oct	Entire	Green‐yellow		S/M	
*P. stenoglossa* Hayata									
*P. stenophylla* Tang & F.T.Wang									
*P. stricta* Lindl.	Hapeman and Inoue (1997), Patt et al. (1989), van der Voort et al. (2022)		North America		Entire	Green‐yellow	Y	S	Yes
*P. taiwanensis* (S.S.Ying) S.C.Chen, S.W.Gale & P.J.Cribb	Efimov (2016)		Asia	Jul–Aug	Entire	Yellow‐green		S	
*P. takedae* Makino	Efimov (2016)		Asia	Jul–Aug	Entire	Yellow‐green		S	
*P. tescamnis* Sheviak & W.F.Jenn.									
*P. tipuloides* (L.f.) Lindl.	Efimov (2016)	LC	Asia	Jul–Aug	Entire	Green‐yellow		S/M	
*P. transversa* (Suksd.) R.M.Bateman	Ackerman (1977)				Entire	Green		S/M	
*P. unalascensis* (Spreng.) Kurtz	Ackerman (1977), Nawrocki et al. (2017), J. K. Janes (2021) (personal observation)		North America	Jun–Aug	Entire	Green		S	Night
*P. undulata* J.J.Sm.									
*P. uniformis* Tang & F.T.Wang	Liden and Bharali (2019)		Asia	Aug–Oct	Entire	White		S	Yes
*P. urceolata* (Hook.f.) R.M.Bateman	Efimov (2016)		Asia	Jul‐Sep	Entire	White		S	Yes
*P. ussuriensis* (Regel) Maxim.	Efimov (2016)		Asia	May–Jul	Entire	Green		S/M	
*P. yadonii* (Rand.Morgan & Ackerman) R.M.Bateman		VU							
*P. yakumontana* Masam.	Efimov (2016)		Asia	July	Entire	White		S	
*P. yangmeiensis* T.P.Lin									
*P. yosemitensis* Colwell, Sheviak & P.E.Moore	Colwell et al. (2007), Lahondère et al. (2020)	EN	North America		Entire	Green		S	Yes
*P. zothecina* (L.C.Higgins & S.L.Welsh) Kartesz & Gandhi		DD							

*Note*: Abbreviations for the International Union for the Conservation of Nature (IUCN) conservation status are: DD, data deficient; EN, endangered; LC,least concern; VU, vulnerable. Full citation details for literature sources can be found in Appendix S1.

**FIGURE 3:**
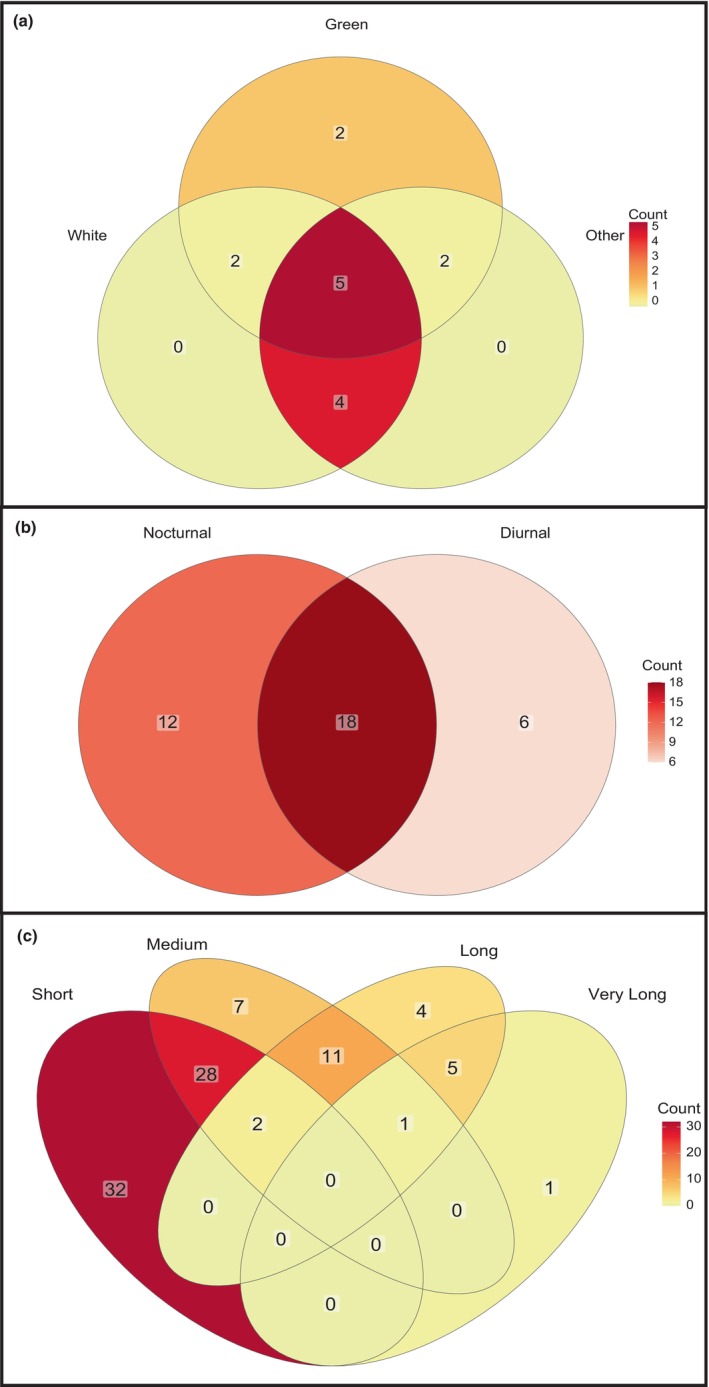
Venn diagrams of flower color (a); timing of floral visitor or pollinator visits (b); and floral spur length categories (c).

Table 1 provides detailed morphological information for a number of *Platanthera* spp. In total, 80 (50.96%) *Platanthera* spp. were described as being green, 44 (28.03%) were described as white, while 48 (30.57%) were described as other colors, such as purple or orange, while 43 (27.39%) species did not have a color description. Many species were placed in multiple categories due to descriptions of greenish‐white or white‐green, for example (Figure 3a). The labella of 83 (52.47%) species were described as entire, whereas 22 (14.01%) species were described as having fringed or dissected labella. Fringed labella were only described for species that occur in North America. There were 52 (33.12%) species without a labellum description.

Using broad categories of spur length (short 1–10 mm, medium 11–20 mm, long 21–30 mm, and very long >31 mm), we found considerable variation among reported lengths for various species, with many species placed in multiple categories. For example, 62 (39.49%) species had small spurs, 49 (31.21%) had medium spurs, 43 (27.39%) had long spurs, and seven (4.46%) had very long spurs (Figure 3c). We could not find definitive measurements for 65 (41.40%) species. Three species (1.91%) were described as having no scent production, 27 (17.20%) were described as emitting a human‐detectable scent, and 127 (80.89%) species had no scent information provided. Among those with a detectable scent, 11 (40.74% of scented species) were described as releasing the scent at night, five (18.52% of scented species) release the scent during the day, and three (11.11% of the scented species) were cited as releasing the scent both day and night. One species (*Platanthera hyperborea*) was described as being scented by one author and non‐scented by another but this may be attributed to changes in taxonomy and known distributions of species – both *P. huronensis* and *P. aquilonis* were previously considered synonymous with *P. hyperborea*.

### 3.3 Pollinator versus visitor

We retrieved >500 records of *Platanthera*–insect associations (Table 2). There were 154 associations among orchids and insects that were specific in stating it was or was not a true pollinator relationship. One association for *P. hologlottis* had contradictory evidence from two different studies (see records for *Thysanoplusia intermixta* in Table 2). One study (Dobson, 1988) cited wrong information from an original source – *P. dilatata* was referenced even though this species was not studied by the original authors (Thien & Utech, 1970). These 154 pollinator accounts span 30 (19.11%) species of *Platanthera*. Seven records state an insect Order as the definitive pollinator, usually citing other papers, but not necessarily the original source, for their information. Two instances, both for *P. lacera*, clearly referred to an insect as a visitor rather than pollinator. Only 92 (59.74%) of the 154 pollinator associations provide additional information pertaining to the time of day (e.g., nocturnal vs. diurnal) during which the interaction was observed, and even fewer provided information about insect behavior and/or pollinia placement on the insect.

**TABLE 2:** Associated floral visitor and pollinator information for various *Platanthera* species obtained from literature sources.

Orchid species	Literature source	Insect order	Insect family	Insect species	Insect IUCN status	Sex of insect	Region of study	Time of day	Insect behavior	Pollinia placement
*P. amabilis* Koidz.	Hapeman and Inoue (1997), Efimov (2016)	Lepidoptera	Noctuidae					Nocturnal		Proboscis
*P. aquilonis* Sheviak	Catling and Catling (1989), Hapeman and Inoue (1997)	Hymenoptera						Diurnal; nocturnal		Proboscis
		Lepidoptera						Diurnal; nocturnal		Proboscis
*P. bifolia* (L.) Rich.	Nilsson (1978), Nilsson (1983a), Maad and Nilsson (2004), Steen and Mundal (2013), Boberg et al. (2014), Esposito et al. (2017), Mõtlep et al. (2018), Brzosko and Bajguz (2019), Trunschke et al. (2020)	Lepidoptera	Erebidae	*Cucullia umbratica*			Belgium	Nocturnal		Proboscis
			Geometridae	*Aplocera plagiata*						
				*Entepheria caesiata*			Sweden; Norway	Nocturnal		
				*Gnophos myrtillata*						
			Noctuidae	*Apamea furva*						
				*Apamea monoglypha*						
				*Autographa gamma*			Belgium	Nocturnal		Proboscis
				*Autographa pulchrina*			Norway	Nocturnal	Grasped flower & fed	
				*Hada nana*						
				*Heliophobus reticulata*						
			Sphingidae				Poland			Tongue
				*Deilephila elpenor*			Estonia			Proboscis
				*Deilephila porcellus*			Sweden; Norway			
				*Hyles gallii*						
				*Sphinx ligustri*			Estonia; Sweden; Norway			Proboscis
				*Sphinx pinastri* (syn. *Hyloicus pinastri*)			Estonia; Norway; Sweden	Nocturnal	Hover & feed	Proboscis; tongue
*P. blephariglottis* (Willd.) Lindl.	Smith and Snow (1976), Cole and Firmage (1984), Catling and Catling (1991), Zettler et al. (1996)	Coleoptera					USA			
		Diptera					USA			
		Hymenoptera	Apidae	*Apis melifera*			USA			
				*Bombus fervidus*	VU		USA			
				*Bombus vagans*	LC		USA			
		Lepidoptera	Danaidae							
			Hesperiidae	*Epargyreaus clarus*			USA			
				*Euphyes ruricola* (syn. *Euphyes vestris*)			USA			
				*Polites coras*			USA			
				*Polites mystic*	LC		USA			
			Lycaenidae	*Strymon melinus*			USA			
			Noctuidae	*Agrotis* sp.			USA			
			Nymphallidae	*Danaus plexippus*	LC		Canada			
			Papilionidae	*Papilio gluacus*						
				*Papilio troilus*			USA	Diurnal	Visits several flowers & lands on labellum	Head
			Pieridae	*Artogeia rapae* (syn. *Lymantria serva*)			USA			
				*Colias philodice*			USA			
			Sphingidae	*Darapsa versicola*			USA	Diurnal	Hovering & probing flowers	
				*Hemaris* sp.			USA			
				*Hemaris thysbe*			USA			
				*Hyles lineata*			USA	Nocturnal		
				*Manduca quinquemaculata*			USA	Nocturnal		
*P. blumei* Lindl.										
*P. boninensis* Koidz.	Inoue (1983), Hapeman and Inoue (1997)	Lepidoptera	Geometridae	*Ascotis selenaria*			Japan			
				*Creora* sp.			Japan			
			Noctuidae					Nocturnal		Proboscis
				*Callopistria maillardi*			Japan			
				*Erceia* sp.			Japan			
*P. chapmanii* (Small) Luer	Folsom (1984), Argue (2012), Poff et al. (2016)	Lepidoptera	Papilionidae	*Papilio* sp.						Proboscis
				*Papilio marcellus* (syn. *Eurytides marcellus*)			USA			
				*Papilio palamades*			USA			
				*Papilio troilus*			USA			
			Pieridae	*Phoebis sennae*	LC		USA			
*P. chiloglossa* (Tang & F.T.Wang) K.Y.Lang


							
*P. chlorantha* (Custer) Rchb.	Nilsson (1978), Nilsson(1983a), Maad and Nilsson (2004), Bateman et al. (2012), Sexton (2014), Esposito et al. (2017), Lindqvist et al. (2018), Mõtlep et al. (2018, Steen et al. (2019)	Lepidoptera	Geometridae					Nocturnal		
				*Aplocera plagiata*			Sweden	Nocturnal		
				*Gnophos myrtillata* (syn. *Gnophos obfuscata*)			Scandanavia			
				*Ourapteryx sambucaria*			Sweden	Nocturnal		
			Hesperiidae	*Ochlodes venata*		M; F	Sweden	Diurnal		
			Noctuidae				Norway	Nocturnal		Eyes; head; labial palps
				*Abrostola tripasia*			Sweden; Scotland; Estonia	Nocturnal; Diurnal		Eyes
				*Apamea* sp.			Sweden			Eyes
				*Apamea anceps*			Sweden	Nocturnal		Eyes
				*Apamea furva*		M; F	Sweden	Nocturnal		Eyes
				*Apamea lateritia*		M; F	Sweden	Nocturnal		Eyes
				*Apamea monoglypha*		M; F	Estonia; Sweden	Nocturnal		Eyes
				*Apamea sublustris*		M; F	Sweden	Nocturnal		
				*Autographa bractea*		M; F	Estonia; Scotland; Sweden	Diurnal; nocturnal		Eyes
				*Autographa gamma*			Belgium; Estonia; Scotland; Sweden	Nocturnal		Eyes
				*Autographa jota*		M; F	Estonia; Scotland; Sweden	Diurnal; nocturnal		Eyes
				*Autographa pulchrina*		M; F	Estonia; Scotland; Sweden	Diurnal; nocturnal		Eyes
				*Cucullia umbratica*		M; F	UK; Belgium; Sweden	Diurnal; nocturnal		Eyes
				*Diachrysia chrysitis*		M; F	Estonia; Scotland; Sweden	Nocturnal		Eyes
				*Diachrysia stenochrysis*			Estonia			Eyes
				*Diarsia mendica*			Sweden	Nocturnal		
				*Hada nana* (syn. *Hada plebeja*)						
				*Hadena albimacula*			Sweden	Nocturnal		
				*Hadena bicruris* (syn. *Hadena azorica*)	CR		Estonia			Eyes
				*Hadena compta*			Sweden	Noctural		
				*Hadena perplexa*			Sweden	Nocturnal		
				*Heliophobus reticulata* (syn. *Sideridis reticulata*)			Sweden	Nocturnal		
				*Noctua pronuba*			Belgium; Estonia; Scotland	Diurnal; nocturnal		Eyes
				*Plusia festucae*			Scotland; Sweden	Diurnal; nocturnal		Eyes
				*Plusia putnami*			Sweden	Nocturnal		
				*Plusia putnami gracilis*			Scotland	Diurnal; nocturnal		Eyes
				*Polia bombycina*			Sweden	Nocturnal		Eyes
				*Polia hepatica*		M; F	Sweden	Nocturnal		Eyes
				*Polia nebulosa*			Sweden	Nocturnal		
				*Rivula sericealis*			Scotland	Diurnal; nocturnal		Eyes
				*Xestia baja*			Estonia			Eyes
			Sphingidae					Nocturnal		
				*Deilephila elpenor*		M; F	Sweden	Nocturnal	Starts at lower flowers & works up	Eyes
				*Deilephila porcellus*		M; F	Estonia; Scotland; Sweden	Diurnal; nocturnal		Eyes
				*Hyles gallii*			Estonia		Starts at lower flowers & works up	Eyes
				*Hyloicus pinastri* (syn. *Sphinx pinastri*)			Norway; Sweden; Estonia	Diurnal; nocturnal	Starts at lower flowers & works up; hover & probe flower	Eyes, head, labial palps
*P. chorisiana* (Cham.) Rchb.f.	Inoue (1983), Catling and Catling (1991)	Coleoptera	Oedemeridae				Japan			
*P. ciliaris* (L.) Lindl.	Smith and Snow (1976), Robertson and Wyatt (1985), Robertson and Wyatt (1990), van der Cingel (2001), Chupp et al. (2015)	Apodiformes	Trochilidae	*Archilochus colubris*	LC		USA			
		Lepidoptera	Lycaenidae	*Strymon liparops* (syn. *Satyrium liparops*)			USA			
			Nymphalidae	*Danaus plexippus*	LC		USA			
			Papilionidae	*Battus philenor*	LC		USA			
				*Papilio glaucus*			USA			
				*Papilio palamedes*			USA	Diurnal		
				*Papilio polyxenes*	LC					
				*Papilio troilus*			USA			Head
			Pieridae	*Phoebis sennae*	LC		USA	Diurnal		Eyes
			Sphingidae	*Hyles lineata*			USA	Nocturnal		
				*Xylophanes tersa*			USA	Nocturnal		Eyes
*P. cristata* (Michx.) Lindl.	Folsom (1984), Hapeman and Inoue (1997)	Hymenoptera	Apidae	*Bombus pensylvanicus*				Diurnal		Head; eyes
		Lepidoptera						Diurnal		Eyes
*P. dilatata* (Pursh) Lindl. ex L.C.Beck	Kipping (1971), Dobson (1988), Vogt (1990), Boland (1993), Catling and Catling (1991), Larson (1992), van der Voort et al. (2022)	Diptera	Calliphoridae				Canada			
			Culicidae	*Aedes* sp.						
			Syrphidae	*Eristalis* sp.			Canada	Diurnal	Hovered near plant	
				*Eupoedes* sp.			Canada	Diurnal	Hovered near plant	
				*Lapposyrhus lapponicus*			Canada	Diurnal	Hovered near plant	
		Hymenoptera	Apidae	*Bombus* sp.			Canada			Head
				*Bombus flavifrons*	LC		Canada	Diurnal	Probed flowers & removed pollinia	Head
				*Bombus melanopygus*	LC		Canada	Diurnal		
				*Bombus mixtus*	LC		Canada	Diurnal	Probed flowers & removed pollinia	Head
				*Bombus sitkensis*	LC		Canada	Diurnal	Probed flowers & removed pollinia	Head
		Lepidoptera	Geometridae	*Rheumaptera* sp.			Canada	Nocturnal		
			Hesperiidae	*Polites coras*			Canada	Diurnal	Starts at lower flowers & works up	Proboscis
				*Thymelicus lineola*			Canada			
			Noctuidae	*Aletia oxygala*			Canada	Nocturnal		Proboscis
				*Autographa californica*			USA	Nocturnal		Proboscis
				*Autographa flagellum*			Canada	Nocturnal		Proboscis
				*Autographa pseudogamma*			Canada	Nocturnal		Proboscis
				*Cerapteryx graminis*			Canada	Nocturnal		
				*Chrysaspidia putnami* (syn. *Plusia putnami*)			Canada	Nocturnal		
				*Dicestra oregonica* (syn. *Anarta oregonica*)			USA	Diurnal	Probed flowers	Proboscis
				*Hyppa indistincta*			USA	Nocturnal		Proboscis
				*Pesudaletia unipuncta* (syn. *Mythimna unipuncta*)			Canada	Nocturnal		Proboscis
				*Phlogophora iris*			Canada	Nocturnal		Proboscis
				*Pseudothyatira expultrix*			Canada	Nocturnal		Proboscis
				*Rhyacia quadrangular*			Canada	Nocturnal		
				*Syngrapha rectangular*			Canada	Nocturnal		Proboscis
			Notodontidae	*Ichthyura apicalis* (syn. *Clostera apicalis*)			Canada			
			Nymphalidae	*Boloria selene terraenovae*	LC		Canada			
				*Speyeria hydaspe*			Canada	Diurnal	Probed flowers & removed pollinia	Proboscis
			Papilionidae	*Papilio brevicauda*			Canada			
				*Papilio glaucus*			USA			
				*Papilio canadensis*			Canada			
				*Papilio gothica* (syn. *Papilio zelicaon*)	LC					
				*Vanessa cardui*	LC					
			Pterophoridae				Canada	Diurnal		
*P. eleg*ans Lindl.	Ackerman (1977)	Lepidoptera	Noctuidae	*Autographa* sp.			USA			Proboscis
				*Chrysapedia nichollae* (syn. *Plusia nichollae*)						
*P. flava* (L.) Lindl.	Catling and Catling (1991), Light and McConaill (1998), Pace (2020)	Diptera	Culicidae	*Aedes* sp.			Canada			Eye shaft; Proboscis
		Lepidoptera					Canada			Proboscis
			Pyralidae	*Anageshna primordialis*						Proboscis
*P. florentii* Franch. & Sav.	Suetsugu and Hayamizu (2014)	Lepidoptera	Crambidae	*Paratalanta* sp.			Japan			
				*Scopariinae* sp.			Japan			
			Geometridae	*Lampropteryx* sp.			Japan			Eyes
*P. grandiflora* (Bigelow) Lindl.	Stoutamire (1974), Hapeman and Inoue (1997), van der Cingel (2001), Pace (2020)	Lepidoptera	Noctuidae					Nocturnal		Proboscis
				*Autographa ampla*						
			Papilionidae	*Papilio gluacus*			USA			
				*Papilio polyxenes*	LC					
				*Papilio troilus*			USA			
			Sphingidae	*Hemaris* sp.				Diurnal		Eyes
*P. hologlottis* Maxim.	Inoue (1983), Hattori et al. (2020)	Lepidoptera	Hesperiidae	*Ochlodes ochraceus*			Japan	Diurnal		
				*Ochlodes venata*			Japan	Nocturnal		
			Lycanidae	*Lycaeides argyrognomon*			Japan	Diurnal		
			Noctuidae	*Thysanoplusia intermixta*			Japan	Diurnal; nocturnal		
				*Trichoplusia intermixta*			Japan	Nocturnal		
			Nymphalidae	*Neptis sappho*			Japan			
			Pieridae				Japan	Diurnal; nocturnal	Held flower & took nectar	
				*Colias erate*	LC		Japan	Nocturnal		
				*Pieris melete*			Japan	Nocturnal		
			Plusiinae				Japan	Diurnal; nocturnal	Hovered at flower & took nectar	
			Sphingidae	*Macroglossum bombylans*			Japan	Diurnal; nocturnal		
*P. hookeri* (Torr.) Lindl.	Boland (1993), Hapeman and Inoue (1997), Pace (2020)	Lepidoptera	Hesperiidae							
			Noctuidae					Nocturnal		Eyes
*P. huronensis* Lindl.	Catling and Catling (1989), Lahondère et al. (2020)	Diptera	Syrphidae				USA	Diurnal		
		Hymenoptera	Apidae	*Bombus appositus*	LC		USA	Diurnal		Tongue
				*Bombus flavifrons*	LC		USA	Diurnal		Tongue
				*Bombus occidentalis*	VU		USA	Diurnal		Tongue
				*Psithyrus insularis* (syn. *Bombus insularis*)	LC		USA	Diurnal		Tongue
				*Psithyrus suckleyi* (syn. *Bombus suckleyi*)	CR		USA	Diurnal		Tongue
		Lepidoptera	Noctuidae	*Aletia oxygala*			USA			
				*Cucullia intermedia*			USA			
				*Trichordestra dodii*			USA			
			Nymphalidae	*Erebia epipsodea*			USA			
				*Vanessa virginiensis*	LC		USA			
*P. hyperborea* (L.) Lindl.	Thien (1969), Catling and Catling (1991), Hapeman and Inoue (1997), Kropf (2015)	Diptera	Culicidae	*Aedes* sp.			USA			
				*Aedes pullatus*			Canada			
				*Aedes punctor*			Canada			
		Hymenoptera	Apidae	*Bombus jonellus*	DD		Iceland	Diurnal	Landed on labellum & probed flower	
		Lepidoptera	Geometridae	*Xanthorhoe* sp.			USA			
			Noctuidae							Proboscis
*P. integra* (Nutt.) A.Gray ex L.C.Beck	Hapeman and Inoue (1997)							Diurnal		Proboscis
*P. integrilabia* (Correll) Luer	Zettler and Fairly (1990), Zettler et al. (1996)	Coleoptera	Mordellidae	*Mordelia* sp.			USA			
		Diptera	Syrphidae	*Allograpta* sp.			USA			
				*Milesia virginiensis*			USA			
		Hymenoptera	Apidae	*Apis melifera*			USA			
				*Bombus impatiens*	LC		USA			
			Vespidae	*Vespula maculifrons*			USA			
		Lepidoptera	Hesperiidae	*Epargyreus clarus*			USA	Diurnal		Eyes
			Papilionidae	*Papilio glaucus*			USA	Diurnal		Eyes
				*Papilio troilus*			USA	Diurnal		Eyes
			Sphingidae	*Manduca* sp.						
*P. japonica* (Thunb.) Lindl.	Hapeman and Inoue (1997), Suetsugu and Tanaka (2013)	Lepidoptera	Noctuidae	*Thysanoplusia intermixta*			Japan	Nocturnal		
			Sphingidae					Nocturnal		Proboscis
				*Macroglossum stellatarum*			Japan	Diurnal		Proboscis
*P. lacera* (Michx.) G.Don	Stoutamire (1974), Catling and Catling (1991), Hapeman and Inoue (1997), Little et al. (2005)	Diptera	Calliphoridae	*Lucilia* sp.			USA			
		Hymenoptera	Halictidae	*Ladioglossum* sp.			USA	Diurnal		
		Lepidoptera	Noctuidae					Diurnal, nocturnal		Proboscis
				*Allagrapha aerea*			USA	Nocturnal	Hovers in front & visits 2.6 flowers per plant	
				*Anagrapha falcifera*			USA	Nocturnal	Hovers in front & visits 2.6 flowers per plant	
			Sphingidae					Diurnal		Proboscis
				*Haemmorhagis thysbe* (syn. Hermaris thysbe)			USA	Nocturnal		Proboscis
				*Hemaris* sp.				Diurnal, nocturnal		Proboscis
*P. leucophaea* (Nutt.) Lindl.	Sheviak and Bowles (1986), Catling and Catling (1991), Hapeman and Inoue (1997), Ross (2012), Thixton (2017), Bell et al. (2021)	Lepidoptera	Sphingidae					Nocturnal		Proboscis
				*Eumorpha achemon*						Proboscis
				*Eumorpha pandorus*						
				*Lintneria eremitus* (syn. *Sphinx eremitus*)			USA	Nocturnal		Proboscis
				*Manduca sexta*						
				*Xylophanes tersa*						
*P. mandarinorum* Rchb.f.	Inoue (1983)	Lepidoptera	Erebidae	*Miltochista striata*			Japan	Nocturnal		Eyes
			Geometridae	*Alcis angulifera*			Japan	Nocturnal		Eyes
				*Melanthia procellata*			Japan	Nocturnal		Eyes
				*Odontopera arida*			Japan	Nocturnal		Eyes
				*Ourapteryx nivea*			Japan	Nocturnal		Eyes
				*Pingasa aigneri*			Japan	Nocturnal		Eyes
				*Photoscotosia atrostrigata*			Japan	Nocturnal		Eyes
			Noctuidae	*Euplexia albovittata* (syn. *Phlogophora albovittata*)			Japan	Nocturnal		Eyes
				*Xylena fumosa*			Japan	Nocturnal		Eyes
			Pyralidae	*Palpita nigropunctalis*			Japan	Nocturnal		Eyes
			Sphingidae	*Theretra japonica*			Japan			
*P. nivea* (Nutt.) Luer	Hapeman and Inoue (1997)	Lepidoptera								Proboscis
*P. obtusata* (Banks ex Pursh) Lindl.	Twinn et al. (1948), Thien and Utech (1970), Gorham (1976), Catling and Catling (1991), Lahondère et al. (2020), Pace (2020)	Diptera	Culicidae	*Aedes* sp.		F	Canada	Nocturnal		Eyes
				*Aedes aegyptii*			USA			Eyes
				*Aedes campestris*			Canada			
				*Aedes canadensis*			USA			Eyes
				*Aedes cinereus*		F	Canada			Eyes
				*Aedes communis*		F	USA			Eyes
				*Aedes diantaeus*						
				*Aedes excrucians*		F	Canada			Eyes
				*Aedes flavescens*			Canada			
				*Aedes hexodontus*		F	USA			
				*Aedes impiger*			Canada			
				*Aedes increpitus*			USA			Eyes
				*Aedes intrudens*		F	USA			
				*Aedes nigripes*		F	USA			Eyes
				*Aedes punctor*		F	USA			Eyes
				*Aedes riparius*			Canada			
				*Aedes spencerii*			Canada			
				*Aedes sticticus*						
				*Aedes vexans*			USA			
		Lepidoptera	Geometridae	*Hydriomedia renunciata* (assumed *Hydriomena renunciata*)			USA			Eyes
				*Mesoleuca ruficillatat*			USA			Eyes
				*Xanthorhoe abrasaria*			USA			Eyes
				*Xanthorhoe lacustrata*			USA			Eyes
				*Xanthorhoe munitata* (syn. *Xanthorhoe decoloraria*)			USA			
			Pyralidae	*Eudonia lugubralis*			USA			
*P. okuboi* Makino	Inoue (1983)	Lepidoptera	Sphingidae	*Rhagastis trilineata*			Japan	Nocturnal		Proboscis
*P. ophrydioides* F.Schmidt	Inoue (1983)	Lepidoptera	Geometridae	*Deileptenia ribeata*			Japan	Nocturnal		
			Noctuidae	*Apamea hampsoni*			Japan	Nocturnal		
*P. orbiculata* (Pursh) Lindl.	van der Pijl and Dodson (1966), Pace (2020), Berry and Cleavitt (2021)	Apodiformes	Trochilidae	*Archilochus colubris*	LC		USA	Diurnal		
		Hymenoptera	Apidae	*Xylocopa* sp.			USA	Diurnal		
		Lepidoptera	Geometridae							
			Noctuidae	*Autographa ampla*						
				*Diachrysia balluca*						
			Pterophoridae	*Sphenarches anisodactylus*			USA	Diurnal		
			Sphingidae	*Sphinx drupiferarum*						
*P. orbiculata* var. *macrophylla* (Goldie) Luer	Hapeman and Inoue (1997), Berry and Cleavitt (2021)	Lepidoptera	Noctuidae					Nocturnal		Eyes
			Sphingidae							
*P. peichatieniana* S.S.Ying	Efimov (2016)	Lepidoptera								
*P. peramoena* A.Gray	Catling and Catling (1991), Hapeman (1997), Hapeman and Inoue (1997)	Lepidoptera	Nymphalidae	*Danaus plexippus*	LC		USA			
			Papilionidae	*Papilio glaucus*			USA	Diurnal		Eyes
				*Papilio troilus*			USA	Diurnal		
			Sphingidae	*Hemaris diffinis*			USA			
				*Hemaris thysbe*			USA	Diurnal		Eyes
				*Hyles lineata*						
*P. praeclara* Sheviak & M.L.Bowles	Sheviak and Bowles (1986), Hapeman and Inoue (1997), Westwood et al. (2011), Ross (2012), Pace (2020)	Lepidoptera	Sphingidae					Diurnal		Eyes
				*Eumorpha achemon*						Eyes
				*Hyles gallii* (syn. *Hyles euphorbiae*)			Canada			
				*Manduca quinquemaculata*						
				*Paratrea plebeja*						
				*Sphinx drupiferarum*			Canada			
*P. psycodes* (L.) Lindl.	Stoutamire (1974), Pace (2020)	Lepidoptera	Hesperiidae	*Polites mystic*	LC		USA			Proboscis
			Papilionidae	*Papilio polyxenes*	LC			Diurnal		
			Sphingidae	*Hemaris diffinis*				Diurnal		Proboscis
				*Hemaris thysbe*			USA	Diurnal		Proboscis
*P. sachalinensis* F.Schmidt	Inoue (1983), Hapeman and Inoue (1997), Suetsugu and Hayamizu (2014)	Lepidoptera	Crambidae	*Paratalanta* sp.			Japan			
			Noctuidae					Nocturnal		Proboscis
				*Athetis lineosa*			Japan			
				*Polychrysia splendida*			Japan			
				*Sineaugraphe disgnosta*			Japan			
				*Sypna hercules* (syn. *Sypnoides hercules*)			Japan			
*P. sonoharae* Masam.	Hapeman and Inoue (1997)	Lepidoptera	Noctuidae					Nocturnal		Proboscis
*P. sparsiflora* (S.Watson) Schltr.	Kipping (1971)	Lepidoptera	Pyralidae				USA	Nocturnal	Probed flowers, removed pollinia	Proboscis
			Pterophoridae	*Platyptillia* sp.			USA	Nocturnal	Visited flowers	
				*Oidaematophorus* sp.			USA	Nocturnal	Visited flowers	
*P. stricta* Lindl.	Patt (1985), Patt et al. (1989), Lahondère et al. (2020), van der Voort et al. (2022)	Diptera	Culicidae	*Aedes* sp.						
			Empididae	*Anthepiscopus longipalpis*			USA			
				*Empis brachysoma*			USA			Proboscis
				*Empis delumba* (assumed *Empis delumbis*)			USA			Proboscis
				*Empis laniventris*			USA			Proboscis
				*Empis virgata*			USA			Proboscis
				*Rhamphomyia* sp.			USA			
			Syrphidae	*Baccha elongata*	LC		USA			
		Coleoptera	Staphylinidae	*Eusphalerum pothos*			USA			
		Hymenoptera	Apidae	*Bombus flavifrons*	LC		USA		Forage rapidly	Glossa
				*Bombus melanopygus*	LC		USA		Forage rapidly	Glossa
			Halictidae	*Lasioglossum* sp.			USA			
		Lepidoptera	Geometridae	*Anagoga occiduaria* (syn. *Plagodis pulveraria occiduaria*)			USA		Forage slowly; probe multiple flowers	
				*Eupithecia* sp.			USA		Forage slowly; probe multiple flowers	
				*Eustroma fasciata*			USA		Forage rapidly	Eyes
				*Xanthorhoe lagganata*			USA		Forage slowly; probe multiple flowers	
			Prodixidae	*Greya* sp.			USA			Proboscis
			Pterophoridae				Canada	Diurnal		
			Tortricidae				USA			
*P. tipuloides* (L.f.) Lindl.	Inoue (1983), Hapeman and Inoue (1997)	Lepidoptera					Japan			
			Noctuidae					Nocturnal		Proboscis
*P. transversa* (Suksd.) R.M.Bateman	Ackerman (1977)	Lepidoptera	Geometridae	*Thallophaga taylorata*			USA			Proboscis
*P. unalascensis* (Spreng.) Kurtz	Ackerman (1977), Nawrocki et al. (2017), van der Cingel (2001)	Lepidoptera	Geometridae	*Eupithecia* sp.			USA			Proboscis
			Pterophoridae				USA	Nocturnal		Proboscis
				*Oidaemetaphorus* sp.			USA			
				*Platyptillia* sp.			USA			
			Pyralidae				USA	Nocturnal		Proboscis
*P. ussuriensis* (Regel) Maxim.	Inoue (1983), Hapeman and Inoue (1997), Nakase and Suetsugu(2016)	Lepidoptera	Crambidae	*Mabra charonialis*			Japan	Nocturnal		
			Pyralidae					Nocturnal		Proboscis
				*Bocchoris inspersalis*			Japan			
				*Bradina* sp.			Japan			
				*Cnaphalocrocis medinalis*			Japan			
				*Goniorhynchus butyrosa*			Japan			
				*Hedylepta similis* (syn. *Omiodes indicata*)			Japan			
				*Herpetogramma rudis*			Japan			
				*Pagyda quinquelineata*			Japan			
*P. yadonii* (Rand.Morgan & Ackerman) R.M.Bateman	Nawrocki et al. (2017)	Hymenoptera	Apidae	*Bombus* sp.			USA			

*Note*: Abbreviations for the International Union for the Conservation of Nature (IUCN) conservation status are DD, data deficient; EN, endangered; LC, least concern; VU, vulnerable. Insect sex, where provided, is abbreviated as M, male and F, female. Full citation details for literature sources can be found in Appendix S1.

We retrieved 233 unique species of insects interacting with 30 (19.11% of total orchids) *Platanthera* spp. These 233 insects represented 138 genera across 30 different families and six orders. One bird genus was also noted interacting with *P. ciliaris* and *P. orbiculatata*. At the level of Order, Lepidoptera were associated with 43 (27.39%) orchid species, while Hymenoptera and Diptera were associated with 12 (7.64%) and nine (5.73%) orchid species, respectively. From the Lepidoptera, the families Noctuidae, Sphingidae, and Geometridae interact with high numbers of *Platanthera* spp. (22, 17 and 13 species of orchid, respectively). On average, 9.00 (SE = ±1.62) insect species interact with each orchid but the average number of orchids for each insect species is 1.31 (SE = ±0.05), highlighting that each orchid can attract several insects but that relatively few insects are shared across orchid species.

Twenty‐four insect associates of *Platanthera* spp. have been assessed and are listed by IUCN. Of those, 21 are listed as Least Concern; two, *Bombus fervidus* F. and *Bombus occidentalis* Greene (both Hymenoptera: Apidae) are listed as Vulnerable; and one, *Psithyrus suckleyi* (Greene) (Hymenoptera: Apidae) is listed as Critically Endangered. Others, such as *Danaus plexippus* L. (Lepidoptera: Nymphalidae), have substantial conservation concerns despite being listed as Least Concern. Due to the lack of assessments for the vast majority of insect associates of the *Platanthera* – combined with increasing documentation of insect declines (Hallmann et al., 2017; Klink et al., 2020), particularly native pollinator declines (Young et al., 2017) – it is likely that conservation issues are not limited to a small handful of insect species.

### 3.4 Specificity or generality of associations

**FIGURE 4:**
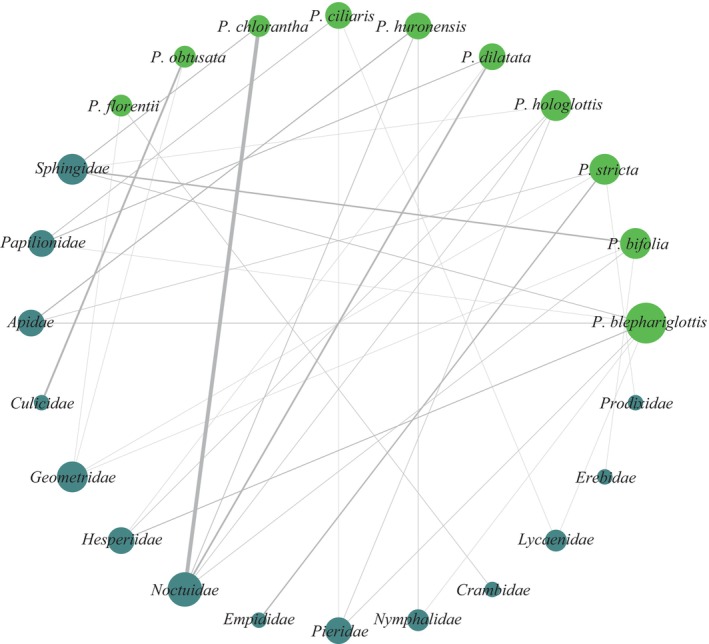
Network showing the extent of sharing among the10 *Platanthera* species with the highest number of floral visitor and pollinator observations at the insect Family‐level. Thicker lines indicate a greater number of observed insect species belonging to that Family. *Platanthera hyperborea* interactions from North America were included under *P. huronensis* because these two represent synonyms – plants previously attributed to *P. hyperborea* in North America are now attributed to *P. huronensis*.

Few *Platanthera* spp. were cited as being highly specific or only associating with one insect species. Just 15 (9.55%) *Platanthera* spp. had five or fewer insect species associated with them. In contrast, *P. chlorantha* had 41 (17.52% of all unique insect records) insect species associated as pollinators or visitors. The greatest Order‐level diversity was associated with *P. blephariglottis*, *P. integrilabia*, and *P. stricta* – each with four (80.00% of the total insect Orders) associated insect Orders. When considering Family‐level associations, *P. dilatata* (11 or 36.67% of all insect Families) and *P. stricta* (10 or 33.33% of all insect Families) had the greater diversity in pollinators/visitors. In total, 20 orchids shared visitors or pollinators, and 43 insects shared at least two *Platanthera* spp. Two sets of six *Platanthera* spp. (*P. blephariglottis*, *P. ciliaris*, *P. dilatata*, *P. grandiflora*, *P. integrilabia*, *P. peramoena* and *P. blephariglottis*, *P. ciliaris*, *P. chapmannii*, *P. grandiflora*, *P. integrilabia*, and *P. peramoena*) were associated with the same visitor or pollinator species (*Papilio glaucus* and *Papilio troilus*, respectively), representing the greatest level of observed pollinator sharing. Figure 4 shows the insect Family‐level associations, and degree of floral visitor and pollinator sharing for the 10 *Platanthera* spp. with the greatest number of insect observations. The average pollinator sharing index was 0.002 across all orchid species.

**FIGURE 5:**
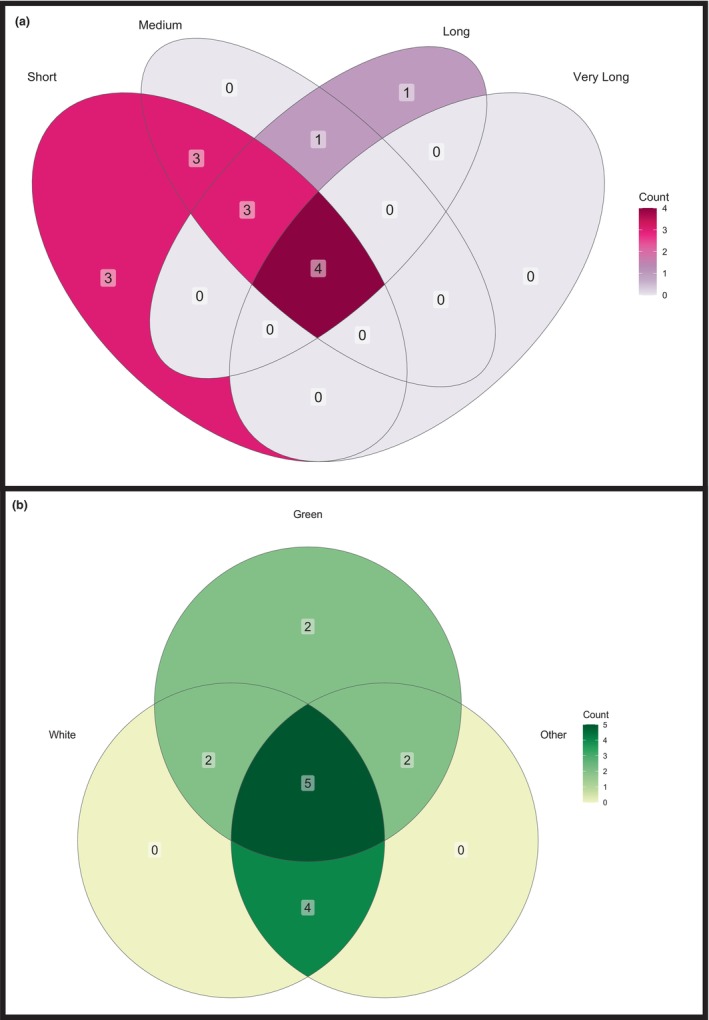
Venn diagrams showing the insect floral visitor and pollinator associations with floral spur length categories (a) and floral color (b).

Five animal Orders were associated with *Platanthera* spp.; one of which was Apodiformes (hummingbirds). Diptera, Hymenoptera, and Lepidoptera were associated with *Platanthera* of all spur length categories. Coleoptera were associated with all spur lengths except the medium category. Twenty‐four insect Families were associated with short‐spurred orchids, 19 with medium spurs, 18 with long spurs, and nine with very long spurs (Figure 5a). Trochilidae (hummingbirds) were only associated with the medium and long spur categories. Seven Families were associated with all spur length categories (Apidae, Geometridae, Hesperiidae, Noctuidae, Papilionidae, Sphingidae, and Syrphidae). Notodontidae, Oedemeridae, Proxidae, Staphylinidae, and Tortricidae were only associated with short‐spurred *Platanthera* spp.

With respect to flower color, we found no pattern at the Ordinal level – every Order of visitor or pollinator was associated with every flower color category. At the Family level, 23 were associated with white flowers, 20 with green flowers, and 22 with other colored flowers (Figure 5b). A total of 13 Families were associated with all flower colors. Four Families (Mordellidae, Notodontidae, Plusiinae, and Vespidae) were associated with white flowers only. However, many of these data points represent single observations. Sphingid and noctuid moths were most commonly associated with white (12 orchid species each) and other colored *Platanthera* spp. (eight orchid species each), while noctuid and geometrid moths were commonly associated with green flowers (16 and 11 orchid species, respectively).

### 3.5 Diurnal versus nocturnal interactions

The time of insect interaction with flowers was available for 122 insects (52.14% of all insect spp.) spanning 36 (22.93%) *Platanthera* spp. (Table 2). All 36 of those *Platanthera* spp. are described as having nocturnal pollination/visitation, 32 (20.38%) had diurnal pollination/visitation, and 10 (6.37%) had both diurnal and nocturnal pollination/visitation (Figure 3b). It is important to note that several *Platanthera* are included in multiple categories because some authors found differences in insect activity in different regions or years. Few literature sources (36/122 useful sources = 29.51%) account for the current knowledge of *Platanthera*–insect interaction timing, and many of those simply cite previous studies.

Nocturnal interactions were reported more frequently than diurnal interactions for all color categories. Green‐flowered species appeared to have more nocturnal interactions (61.1% of all nocturnal interactions), compared to other colors, but these data were not significant according to a Fisher exact test (white vs. green two‐tailed *p*‐value = .61; green vs. other two‐tailed *p*‐value = .42). Of the 27 *Platanthera* spp. reported as having a scent, nocturnal floral scent production (11/27 = 40.74%) was observed more frequently than diurnal scent production (5/27 = 18.52%), but true tests to assess scent production time were not performed.

### 3.6 Linking pollinator needs and behavior to orchid reproductive success

Descriptions of the behavior exhibited by the visitor or pollinator were available for only 13 orchid species (8.28%). The most commonly reported behaviors include hovering near and probing flowers and probing flowers from the bottom to the top of the inflorescence spike. A total of 42 (26.75%) *Platanthera* spp. had descriptions of where the pollinia were placed on the vector – all of which were concentrated around the head. The eyes and proboscis were the most commonly cited areas for pollinia placement on insect visitors/pollinators. Only records for *P. chlorantha* and *P. obtusata* provided information on insect pollinator or visitor sex. Of the few studies that reported the amount of nectar present, it seems that nectar quantity is highly variable, with reports of 2–4 mm in the spur (Nilsson, 1978) and 3.3 mg (Stpiczynska, 2001) for *P. chlorantha*, 20 μL (Travers et al., 2018) for *P. praeclara*, and 0.1 μL (Patt et al., 1989) for *P. stricta*. *Platanthera* nectar typically has a high sugar concentration, and sucrose concentrations are much higher than fructose or glucose, according to the few sources available. Sources indicate the following concentrations: 85.8% sucrose, 7.2% glucose, and 7% fructose (Lindqvist et al., 2018) and 599 mM of sugar (Brzosko & Bajguz, 2019) in *P. bifolia*; 70% sucrose, 16.4% glucose, and 13.5% fructose (Lindqvist et al., 2018) and 842 mM of sugar (Brzosko & Bajguz, 2019) for *P. chlorantha*; 18.9% sugar for *P. integrilabia* (Zettler et al., 1996); 24.7% sugar for *P. praeclara* (Westwood et al., 2011); and 8% sugar for *P. stricta* (Patt et al., 1989). Information relating to scent chemistry, floral fertilization and fecundity, and pollinia removal rates was typically lacking from the reviewed studies. A few studies report high amounts of benzenoids (Trunschke et al., 2020), linalool (Nilsson, 1983a, 1983b), lilac aldehyde (Esposito et al., 2018; Lahondère et al., 2020), and caryophyllene (Nilsson, 1978) for *P. bifolia*, *P. chlorantha*, *P. huronensis*, and *P. obtusata*.

## 4 DISCUSSION

To better understand the current state of knowledge relating to the 157 *Platanthera* spp., and their pollinators, we read 297 unique literature sources, of which 122 provided useful information. In spite of *Platanthera* being a widespread and species‐rich genus, many *Platanthera* spp. lack basic distribution and other natural historical information, and relatively few species (19.11%) have floral visitor or pollinator information. While many literature sources seem to provide visitor/pollinator information, it is often information cited from an older study, with few new studies investigating the pollination ecology of *Platanthera* spp. Below, we discuss our results in more detail and provide some recommendations.

### 4.1 General *Platanthera* information and trends

Approximately 50% of terrestrial orchids are considered at‐risk of extinction (Swarts & Dixon, 2009). For *Platanthera* spp., 12.74% of species are given an IUCN status. This is likely an underestimate of the number of species of conservation concern as it does not take into account national‐ or local‐level policies. In addition, many species, particularly those present in Asia, and hybrid species have not been studied enough for robust conservation recommendations to be made. North America is often cited as a centre of diversity for *Platanthera* spp. (Wettewa et al., 2020; Wettewa & Wallace, 2021). However, from a continental distribution perspective, Asia appears to be the diversity hotspot. This may suggest that *Platanthera* sensu lato has Asian origins, or it may simply reflect differential rates of colonization, radiation, and extinction throughout a previously cosmopolitan distribution. Interestingly, all species with fringed labella were described as having North American distributions, and flower colors other than green or white were associated with North American and Asian distributions only. These observations add more weight to the ideas that there are differential rates of colonization, radiation, and extinction and suggest that continental patterns in insect diversity influence *Platanthera* spp. (e.g., Stebbins most efficient pollinator principle (Stebbins, 1970)). It is difficult to make solid statements about worldwide *Platanthera* diversity because of uneven sampling and reporting. For instance, there were no literature records of *Platanthera* spp. in South America, but there are observations attributed to *Platanthera* spp. there on public databases that need to be verified. In addition, sampling and reporting within continents (e.g., Africa) are often clustered so as to provide minimal information on true distributions.

Flower morphology is often linked to pollination syndromes and levels of pollinator specificity (Gravendeel et al., 2004) even though attempts to group co‐associated traits may represent an over‐simplification (Ollerton et al., 2009). Spur length was often cited as a strong reproductive isolation mechanism in *Platanthera* spp. because particular insect taxa are better suited to acquire nectar from particular flower spurs given the length of their proboscis (Boberg et al., 2014; Chapurlat et al., 2015), although other floral features like distance between anthers can play a role (see “Specificity or generality of associations” in the discussion), and some authors suggest that spur length variation is driven largely by climate (Bateman & Sexton, 2008). Scent is another important cue that helps attract certain pollinators or other insect visitors. For example, the timing with which scent is released, and the chemical composition of the scent can lead to effective reproductive isolation among species and populations (Brand et al., 2020; Gaskett, 2011). Thus, we summarized morphological information using broadly defined categories but note that there is considerable variation among observations suggesting that these floral traits may be locally adapted to pollinators (Boberg et al., 2014). A high proportion of species were described as having short spurs (39.49%), adding support to the hypothesis that the ancestral form for *Platanthera* spp. was short spurred, and that spur length has been increasing over time (Hapeman & Inoue, 1997). A high number of species were identified as emitting a scent at night, again supporting the idea that short‐tongued nocturnal moths were the likely pollinators of the ancestral *Platanthera* form (Hapeman & Inoue, 1997). However, the knowledge gaps for a considerable number of species (e.g., 41.40% of species have no spur measurements; 80.89% of species have no reported scent information) make it difficult to conclude much with certainty.

### 4.2 Pollinator versus visitor

Considering the ecological importance of pollination, it is surprising that there is such limited information for many plant–pollinator interactions in this group. There were accounts of plant–pollinator interactions for just 19.11% of known *Platanthera* spp. Nearly all of those interactions involved insects – just one bird species was observed. Many sources did not make a clear distinction between visitation and pollination. Several factors contribute to the challenge of classifying specific relationships, including the sheer numbers of orchid‐insect species interactions, accurate identification of insects and orchids, geographic and temporal variation in interactions, the unreliable nature of flowering (i.e., terrestrial orchids can remain dormant for several years (Reddoch & Reddoch, 2007)), and the relatively short flowering times (i.e., flowers may last as little as 7 days (Stpiczynska, 2003)). To successfully conserve orchid and associated insect populations, we must develop detailed databases of the known *Platanthera*‐insect relationships across both time and space; pollination network studies may be more successful.

The distinction between visitor and pollinator typically requires that observers document: (1) transfer of pollen onto an insect, (2) subsequent movement of pollen by the insect, (3) transfer of pollen from the insect to the stigma (i.e., pollination), and (4) that pollination results in fertilization (i.e., pollen tube growth to the ovaries) (Cox & Knox, 1988). Complicating the distinction further, additional orchid‐specific conditions were listed by van der Cingel (2001), including: (5) floral rewards must be shown to be consumed or otherwise used by the insect, (6) variation in floral morphology must be demonstrated to attract the insect, (7) contributions of pollen versus ovules to the next generation must be the result of pollination processes, (8) inter‐relations of multiple vectors must be demonstrated at the community level, and (9) in the case of pseudocopulation and shelter rewards, sexual stimulation by scent, and sleeping in flowers must be demonstrated, respectively. Theoretically, this list of conditions provides a robust framework for distinguishing anything from casual visitors to true pollinators. However, the practicality of obtaining data to meet these conditions is often a limiting factor.

Many authors continue to equate floral visitors with true pollinators (e.g., Steen and Mundal (2013)), or equate pollen transfer and movement by a vector as definitive orchid pollination (e.g., Esposito et al., 2017; Smith & Snow, 1976), without confirming the other necessary conditions. While these are convenient proxies, without experimentation directed at proxy accuracy, the number of true pollinators for each *Platanthera* spp. may be inflated. For instance, Hattori et al. (2020) photographed floral visitors and then inferred fertilization rates from pollinia removal and capsule development among non‐bagged and bagged‐plants, but this is not a direct link to a specific pollinator and its efficacy in pollination or fertilization (or even abiotic factors). The current bottlenecks for *Platanthera* spp. pollination studies appear to be in observing pollinia removal and deposition, linking pollination with fertilization, and demonstrating that rewards and morphology influence pollinators.

### 4.3 Specificity or generality of associations

Several sources have observed that *Platanthera* spp. are primarily pollinated by Lepidoptera, particularly noctuid and pyralid moths (Bateman et al., 2013; Boland, 1993; Schiestl & Schlüter, 2009). Additional authors have hypothesized that only long‐tongued insects would be able to obtain nectar rewards from species with longer floral spurs (Bateman et al., 2013; Chupp et al., 2015). Our literature review confirms that noctuid, sphingid, and geometrid moths are the most common Lepidoptera associated with *Platanthera* spp., irrespective of spur length.

The state of knowledge for *Platanthera* pollination ecology is low compared to some other groups of orchids. For instance, a review of Euro‐Mediterranean orchids revealed that 243 of 512 (47.46% vs. the 19.11% reported here for *Platanthera* spp.) known orchid species in the region have known pollinators, and that 773 insect species interact with those 243 orchids, representing an average of 3.18 insects per orchid species (Schatz et al., 2020). That review identified 87 interacting insect Families across four Orders (Schatz et al., 2020). Our review covered a much broader geographic range, which may explain why we found slightly more insect Orders (i.e., five). Although we considered fewer orchid (259) and insect (233) species, the average number of insects per orchid species (9.00) was found to be considerably higher than that observed for orchids in multiple genera across the Euro‐Mediterranean (3.18). The pollinator sharing index calculated here (0.002) was two orders of magnitude lower than the 0.46 value reported for *Platanthera* spp. by Schiestl and Schlüter (2009). These results indicate that *Platanthera* spp. generally receive a more diverse assemblage of visitor or pollinator partners in comparison to some other orchid groups, but the number of shared pollinators is low suggesting that effective reproductive isolation may exist among some sympatric species. Twenty *Platanthera* spp. shared visitors or pollinators, with two sets of six *Platanthera* spp. sharing a single insect visitor or pollinator (*Papilio glaucus* and *Papilio troilus*). Linkages such as these may be increasingly important to identify. Identification of shared pollinators will allow for more successful reintroductions because informed pollinator surveys will allow predictions of successful reproduction, although mycorrhizal symbionts (Wright et al., 2015) and unintended hybridization events would also need to be considered.

Many authors have attempted to identify suites of floral traits that can be used to generalize or predict the pollinators of certain flowering plants (Fenster et al., 2004; Vereecken et al., 2010). Sources advocating for predictive pollination syndromes indicate that spur length, flower color, and flower size are the most important traits impacting pollinator behavior (Bateman et al., 2013; Hapeman & Inoue, 1997; Schiestl & Schlüter, 2009). For example, the orange flowers and long spur of *P. ciliaris* were expected to be pollinated by diurnal, long‐tongued butterflies (Chupp et al., 2015). Catling and Catling (1991) predicted that white flowered species with long spurs, fringed labella, and scent released at night should be pollinated by large moths, while green flowers with short spurs should be pollinated by other Lepidoptera and other groups. Such predictions provide a starting point for further experimental work but provide little useful information in the interim as both morphological groups are effectively pollinated by Lepidoptera. Additionally, work on *P. lacera* shows that spur length variation is not under selection from its primary pollinator (Little et al., 2005).

The findings from our review contradict some long‐held beliefs about *Platanthera* pollination syndromes – specifically the assertions that green‐flowered plants are primarily pollinated by pyralid and noctuid moths; white‐flowered plants by noctuid and sphingid moths (Hapeman & Inoue, 1997). We found that geometrid and noctuid moths are most commonly associated with green flowers, and that while noctuids and sphingids (both are known to associate with eight *Platanthera* spp.) are often associated with white flowers, they are also commonly associated with non‐white flowers. *Platanthera grandiflora* is presented as having a pollination syndrome suited to diurnal sphingids (Hapeman & Inoue, 1997), whereas our review shows that *Papilio* spp. (Papilionidae) are more often associated with its flowers. Further, other work states that scent can be a more powerful cue than visual stimuli for some insect groups (Inoue, 1985). Such examples serve as a good reminder that while pollination syndromes may be useful generalizations, they should not be automatically equated as representing the full diversity or complexity of possible relationships.

Pollination syndromes are also confounded by spatial and temporal variation. We found considerable variation in reported spur lengths. For example, *P. chlorantha* spurs were reported as 20 mm (Maad & Nilsson, 2004) and as 32 mm (Stpiczynska, 2001). Such differences suggest that regional and temporal climatic conditions, genetic diversity, and local pollinator abundances may drive local selection in spur length. Some populations may experience different selection pressures depending on what their local pollinator is, its relative efficacy in transporting pollen, and its local density. Indeed, some authors have noted substantial ecotype variation in response to the longest‐tongued insect in the area (Robertson & Wyatt, 1990). In contrast, a study of Eurasian *Platanthera* spp. suggested that spur length may be better explained by latitudinal gradient and environment rather than by pollinators (Bateman et al., 2013), and a Scandinavian study showed that spur length variation was explained solely by abiotic conditions (Boberg et al., 2014). The low numbers of *Platanthera* spp. in longer spur length categories suggest that shorter spurs are likely to have been the ancestral state for this character, as indicated by Hapeman and Inoue (1997).

The ancestral placement of pollinia on vectors is believed to be the tongue or proboscis, with more‐derived *Platanthera* spp. attaching pollinia to the eyes of pollinators (Hapeman & Inoue, 1997; Maad & Nilsson, 2004). Just 26.75% *Platanthera* spp. had descriptions of where the pollinia were placed on the body of the vector – all of which were concentrated around the head. This is an expected pattern considering the structure of the flowers; vectors must insert mouthparts toward the spur at the back of the flower in order to obtain nectar. Slight differences in column structure are believed to drive specificity and reproductive isolation in many orchid species (Bateman et al., 2013; Catling & Catling, 1991; Schiestl & Schlüter, 2009). Indeed, subtle changes in column width are believed to be primary mechanism for increasing reproductive isolation among *P. chlorantha* and *P. bifolia* (Schiestl & Schlüter, 2009) and *P. psycodes* and *P. grandiflora* (Stoutamire, 1974). In this context, it is surprising that we found 29 insect species interacting with *P. chlorantha*, all with pollinia placement on the eyes. In contrast, Schiestl and Schlüter (2009) suggest that increasing pollinator specialization may simply be linked to orchid population density – smaller populations need to ensure maximal pollination rates so they tend toward increasingly specialized relationships.

These data highlight considerable knowledge gaps as the specifics of many *Platanthera–*insect relationships remain unknown. Much of the data presented here appear to reflect bias in the *Platanthera* species studied. For example, *P. bifolia* and *P. chlorantha* appear to be very popular research subjects, presumably because many researchers have been interested in understanding the hybridization events that occur between them. Thus, the specific research questions being addressed will clearly influence how much plant‐insect data will be collected in any given study. Underestimates of visitor or pollinator numbers are likely for difficult‐to‐identify groups, such as microlepidoptera and small Diptera. For example, a review of moth‐orchid interactions across North America revealed 227 unique interactions representing just 129 species from seven Families – microlepidoptera, as a group, accounted for 25% of these interactions but there were few identifications to lower taxonomic level (Hahn & Brühl, 2016). Further, few studies incorporate nocturnal investigations, and potential pollinator data are often recorded opportunistically rather than being a formal component of the study.

### 4.4 Diurnal versus nocturnal interactions

Few sources specifically described the timing of the insect interaction. Those studies that mentioned observation time indicated nocturnal activity. Interestingly, few *Platanthera* spp. are expected to be diurnally pollinated as it is believed that the ancestral flower color is green, which is said to attract nocturnal moths (Hapeman & Inoue, 1997). Contrary to the expected patterns based on pollination syndrome theory, we found the reported numbers of *Platanthera* spp. with nocturnal versus diurnal interaction to be similar. The number of nocturnal interactions was greater than diurnal interactions across all flower color categories, although green‐flowered species had slightly higher (nonsignificant) nocturnal interactions compared to white and non‐white flowers. Other authors have commented that *Platanthera* spp. with “other” colored flowers and fringed labella tend to be diurnally pollinated (Catling & Catling, 1991).

Nocturnal pollination has been hypothesized as facilitating highly specialized plant–pollinator relationships and subsequent reproductive isolation (e.g., one moth species to one orchid species) (Macgregor & Scott‐Brown, 2020). A recent review of nocturnal pollination found that highly specialized pollinator–plant relationships are rare, primarily because most moths appear to be generalist nectivores (Macgregor & Scott‐Brown, 2020). However, many authors argue that even when orchids are making use of nonspecific pollinators, reproductive isolation may still be strong owing to differential placement of pollinia on the body of the pollinator (Catling & Catling, 1991; Efimov, 2016; Esposito et al., 2017). Several sister taxa appear to be functionally isolated due to differential pollinia placement on a shared pollinator (e.g., *P. bifolia* and *P. chlorantha*, *P. leucophaea*, and *P. praeclara*) in some parts of their range but not others (e.g., reports of *P. bifolia* and *P. chlorantha* hybrids) (Efimov, 2016; Nilsson, 1983a, 1983b). Thus, the link between nocturnal pollination and increasing plant–insect specialization seems weak. However, with limited research across *Platanthera* spp. and their ranges, it is difficult to make conclusive statements.

It is important to address the knowledge gaps relating to known pollinators and the timing of their activity. The management of orchids and their habitat should include best practices for managing a diversity of pollinators. For example, in a study on *P. bifolia* and *P. chlorantha* pollinator moth diversity was higher in taller vegetation but the orchids preferred shorter and less dense vegetation (Mõtlep et al., 2018). Some insect species appear to be experiencing local declines (e.g., Wagner et al., 2021), and many moth species in particular experience temporal and spatial variation in population density (Hahn & Brühl, 2016). In North America alone, there have been declines in eight species of Sphingidae and Saturnidae attributed to light pollution, forest clearing, and increases in parasitoid populations (Young et al., 2017).

Understanding the types of pollinators interacting with certain orchid species will improve management of orchid demographics and genetic diversity. For instance, there can be variation in the dispersal and foraging behaviors between diurnal and nocturnal insect species. The diurnal *Aporia crategi* (Lepidoptera, Pieridae) was observed moving greater distances than the nocturnal *Zygaena minos* (Lepidoptera, Zygaenidae), both of which pollinate *Anacamptis pyramidalis* (Orchidaceae, Orchidoideae) (Vereecken et al., 2010). Thus, diurnal and nocturnal insects may contribute to pollinia dispersal, and subsequent gene flow, in different ways which may also impact the relative rates of selection on floral traits. Knowledge of the behavioral and ecological differences between and among diurnal and nocturnal pollinators will further our understanding of, and management for, pollinator limitation – a common concern for many orchid populations (Chupp et al., 2015; Young et al., 2017).

Finally, to appreciate the intrinsic value of biodiversity (Rea & Munns Jr, 2017) and to understand the co‐evolutionary relationships among plants and insects, we need to know what insects perform pollination services for what plants and how these patterns may shift over time and space. Pollinator–plant interactions can occur across substantial geographic ranges with differing evolutionary processes in different locations depending on the various biotic and abiotic factors that are present. The concept of a geographic mosaic of co‐evolution (Gomulkiewicz et al., 2000; Thompson, 1999, 2005) suggests that plant species with adjacent or overlapping ranges that interact with a suite of pollinators – as is the case for many *Platanthera* spp. – will have different and complex co‐evolutionary outcomes. This is made more complex by the fact that many pollinators are generalists that are co‐evolving with other non‐*Platanthera* spp. Thus, evolutionary processes acting on *Platanthera—*insect interactions are the result of both co‐evolutionary processes at that site and potentially sites and species much further away. Studying *Platanthera* spp. pollination in the context of co‐evolutionary geographical mosaics (e.g., Johnson & Anderson, 2010) would shed further light on this concept and inform plant and insect conservation efforts.

### 4.5 Linking pollinator needs and behavior to orchid reproductive success – future research

Identifying co‐evolutionary selective pressures among plants and insects is important but challenging. Botanists and entomologists often use different approaches – botanists may focus on the impacts of pollination on reproductive success (Inoue, 1986; Vojtkó et al., 2015), gene flow (Wallace & Bowles, 2023), and population dynamics (Berry & Cleavitt, 2021), whereas entomologists may focus on the energetics of obtaining a reward or trying to fly with a pollen load, synchrony among plant and pollinator phenology, or attempts to cheat the system through nectar robbing (Feuerbacher et al., 2003; Fox et al., 2015; Mountcastle et al., 2015; Slominski & Burkle, 2021). Efforts to study both the behavior and energetic needs of pollinators and the subsequent consequences on reproductive output in plants, simultaneously are not always common. Yet it is clear that disruption to mutualistic relationships among plants and animals can have extreme impacts on population dynamics, local extinction rates, and other evolutionary processes (Chupp et al., 2015; Gaskett, 2011). More integrated approaches that consider the interaction from all directions are needed.

While data are sparse, some general patterns are present from the literature. Many of the interactions involved the pollinators beginning by probing flowers at the bottom of the inflorescence and working toward the top. This behavior seems consistent with the limited studies of floral maturation and nectar production for *Platanthera* spp. where flowers open from the base of the inflorescence toward the top, and nectar production begins approximately 2 days before the flowers open (Stpiczynska, 2001, 2003). Nectar availability was lower in younger flowers (Stpiczynska, 2001, 2003). Similar patterns of flower maturation in *P. stricta* were observed by Patt et al. (1989) with 70–90% of flowers opening acropetally within 2 weeks. In *P. praeclara*, nectar levels and sugar concentrations gradually decline over the flowering period, but sugar concentrations are typically higher in flowers lower on the inflorescence (Westwood et al., 2011). Pollinated flowers continue to provide nectar for several days (Stpiczynska, 2003). These patterns suggest resource allocation trade‐offs in which older flowers, which may be pollinated earlier, receive more investment and continue to attract pollinators so that younger, possibly less attractive and rewarding flowers, at the top of the inflorescence receive pollen too.

Few descriptions of insect behavior at or on the plants are provided in spite of the potential for this to influence pollination efficacy. Landing on the labellum is believed to be the ancestral pollination mechanism (Hapeman & Inoue, 1997). It is likely that hovering behaviors are more energetically expensive (Brzosko & Bajguz, 2019; Campos et al., 2015). If this is true, we might expect hovering behaviors to be associated with long‐spurred flowers as they may provide greater quantities of nectar. However, of the *Platanthera* spp. with clear references to hovering behaviors by their pollinators, the literature indicates that they have variable spur lengths. Similarly, few studies have considered how weather can impact the behavior of insect pollinators, and the consequence such behavioral shifts may have on orchid reproductive success (e.g., Friesen & Westwood, 2013; Maad, 2000; Maad & Alexandersson, 2004).

Studies of nectar composition are rare even though nectar nutritional content could affect visitation length and pollinator behavior. For example, bees prefer high sugar concentrations (35%), whereas hawkmoths prefer lower concentrations (19%) (Brzosko & Bajguz, 2019). The few studies reporting sugar concentration or composition for *Platanthera* spp. show considerable variation – *P. praeclara* nectar is 25% (Fox et al., 2013; Westwood et al., 2011) and *P. stricta* is 8% (Patt et al., 1989) sugar. The concept of pollination syndromes suggests that *P. stricta* must be pollinated by something other than bees or hawkmoths given the low concentrations of sugar. However, several bees are cited as pollinators for this species (Patt et al., 1989).

There is a growing body of evidence of three‐way interactions among pollination vectors, nectar microbiome, and fitness impacts on plants (Chappell & Fukami, 2018; Jacquemyn et al., 2013; Tsuji & Fukami, 2018). However, few studies have specifically investigated the chemical or microbial composition of *Platanthera* nectar, and few links are made among plant investment in nectar relative to pollinator specificity and subsequent pollination efficacy. Processes such as nectar microbes influencing the composition, quantity and volatiles of nectar, which have been identified in other plant species (Toju et al., 2018; Vannette & Fukami, 2018), may be occurring in *Platanthera* spp. and could reinforce or breakdown pollinator specificity, for example. For the few *Platanthera* spp. for which scent profiles have been developed, it is clear that species and populations exhibit considerable variation (Nilsson, 1983b; Steen et al., 2019), and there is evidence to suggest that feeding on nectar can stimulate chemical cues in *P. chlorantha* and *P. bifolia* to attract more insect pollinators (Nilsson, 1983b). These interactions are rich with opportunities for basic chemical ecological investigations that could result in actionable conservation recommendations.

Information on pollinator foraging and dispersal distances and on the sex of pollinators is also sparse in the literature. Because movement of pollinia represents a substantial contribution to gene flow, an understanding of pollinator foraging behavior and dispersal distances is important in understanding orchid population structure, demography, the relative contribution between male and female fitness, and the likelihood of translocation or reintroduction success. For example, variation in habitat can change the primary pollinator resulting in different foraging distances (Vereecken et al., 2010). Relative visitation rates between male and female visitors/pollinators may be an important gap to fill as differences among the sexes in foraging and dispersal distances are known for many insects (Smith et al., 2019). Differences in the average proboscis length between males and females have also been noted – males have a longer proboscis and may be more likely to engage in nectar robbing than actual pollination (Chupp et al., 2015). In such situations, the density and sex ratio of insect populations is an important conservation consideration.

## 5 CONCLUSION

In this review, we have provided the most recent and comprehensive information for plant–animal interactions within the genus *Platanthera*. While many studies mention associations among orchids and insects, relatively few studies have added new information on the natural history of those interactions. We also note that there is missing information for many species, while other species are commonly studied – but generally on small spatial scales. The application of pollination syndromes to describe and predict interactions among *Platanthera* spp. and insects is complex and some generalizations appear to hold merit while others do not. Overall, we advise against the use of pollination syndromes to characterize *Platanthera* spp. pollination. Over time, more data from more locations have shown that *Platanthera* spp. appear to be generalists in terms of their interactions with pollinators (so far known to be mostly insects) – each orchid species associating with several insect species and displaying considerable spatial and temporal variation. More studies should focus on understanding the diversity of associations, and their spatial and temporal patterns, because *Platanthera* spp. offer the opportunity to better understand the ecological and evolutionary mechanisms contributing to floral diversification and co‐evolution – and for better conservation of plant and insect populations.

## AUTHOR CONTRIBUTIONS

**Jasmine K. Janes:** Conceptualization (lead); investigation (lead); visualization (supporting); writing – original draft (lead); writing – review and editing (lead). **Genevieve E. van der Voort:** Investigation (supporting); visualization (lead); writing – original draft (supporting); writing – review and editing (supporting). **Dezene P. W. Huber:** Investigation (supporting); writing – original draft (supporting); writing – review and editing (supporting).

## Supporting information


Appendix S1.

## Data Availability

All data are provided in this publication and the associated appendices.
